# The Effect of LPS and Ketoprofen on Cytokines, Brain Monoamines, and Social Behavior in Group-Housed Pigs

**DOI:** 10.3389/fvets.2020.617634

**Published:** 2021-01-07

**Authors:** Christina Veit, Andrew M. Janczak, Birgit Ranheim, Judit Vas, Anna Valros, Dale A. Sandercock, Petteri Piepponen, Daniela Dulgheriu, Janicke Nordgreen

**Affiliations:** ^1^Department of Paraclinical Sciences, Faculty of Veterinary Medicine, Norwegian University of Life Sciences, Oslo, Norway; ^2^Department of Production Animal Clinical Science, Faculty of Veterinary Medicine, Norwegian University of Life Sciences, Oslo, Norway; ^3^Department of Animal and Aquacultural Sciences, Faculty of Biosciences, Norwegian University of Life Sciences, Ås, Norway; ^4^Research Centre for Animal Welfare, Department of Production Animal Medicine, University of Helsinki, Helsinki, Finland; ^5^Animal and Veterinary Science Research Group, Scotland's Rural College, Roslin Institute Building, Easter Bush, Midlothian, United Kingdom; ^6^Division of Pharmacology and Pharmacotherapy, Faculty of Pharmacy, University of Helsinki, Helsinki, Finland

**Keywords:** lipopolysaccharide (LPS), ketoprofen, social behavior, sickness behavior, cytokines, kynurenine, tryptophan, monoamines

## Abstract

Poor health is a risk factor for damaging behaviors, but the mechanisms behind this link are unknown. Injection of pigs with lipopolysaccharide (LPS) can be used to model aspects of poor health. Recent studies have shown that LPS-injected pigs perform more tail- and ear-directed behavior compared to saline-injected pigs and suggest that pro-inflammatory cytokines may play a role in these behaviors. The aims of this study were to test the effect of LPS on the social behavior of pigs and the neurotransmitters and modulators in their brains and to test the effect of a nonsteroidal anti-inflammatory drug on the effects of LPS. Fifty-two female pigs (11–12 weeks) were allocated to four treatments comprising two injections: saline–saline (SS), saline–LPS (SL), ketoprofen–saline (KS), and ketoprofen–LPS (KL). Activity was scan-sampled every 5 min for 6 h after the last injection in the pen. Social behavior was observed continuously in 10 × 15-min bouts between 8 a.m. and 5 p.m. 1 day before (baseline) and 1 and 2 days after the injection. Saliva was analyzed for cortisol and plasma for tryptophan and kynurenine. The frontal cortex, hippocampus, hypothalamus, and brain stem were sampled 72 h after the injection and analyzed for cytokines and monoamines. LPS activated the HPA axis and decreased the activity within 6 h after the injection. Ketoprofen lowered the effect of LPS on cortisol release and attenuated the behavioral signs of sickness in challenged pigs. SL pigs manipulated the ears of their pen mates significantly longer than SS pigs 2 days after the injection. LPS had no observed effect on IFN-γ, TNF-α, and IL-18. At 72 h after the injection, plasma tryptophan was depleted in SL pigs, and tryptophan and kynurenine concentrations in the frontal cortex and brain stem of SL pigs were significantly lower compared to those in SS pigs. Dopamine concentrations in the hypothalamus of SL pigs were significantly lower compared to those in SS pigs. Serotonin concentrations in the hypothalamus and noradrenaline concentrations in the hippocampus of SL pigs were significantly lower compared to those in KL pigs. In conclusion, LPS influenced the different neurotransmitters and modulators in the brain that are hypothesized to play an important role in the regulation of mood and behavior.

## Introduction

Sickness in humans and other mammals affects many aspects of behavior, including failure or inability to participate in subsistent activities and social interactions due to malaise, pain, and weakness (1). Typically, sick individuals employ health-restoring and rehabilitative strategies such as the avoidance of activity, conservation of energy, limiting of social interactions, reduction of food intake, and seeking rest. In commercial pig production systems, animals housed in close confinement cannot withdraw from their pen mates when they experience a bout of illness, and this might influence their social interactions. The behavioral components of sickness represent, together with the fever response and the associated neuroendocrine changes, a highly organized strategy of the organism to fight an infection [reviewed by Konsmann et al. (2)]. Infectious microorganisms that invade the body encounter, as a first line of defense, macrophages that express toll-like receptors (TLRs). They bind to extracellular pathogen-associated molecular patterns such as lipopolysaccharide (LPS), a cell wall component of Gram-negative bacteria, and initiate the transcription and release of pro-inflammatory cytokines into the blood stream (3). Of these cytokines, mainly interleukin 1-β (IL1-β), interleukin 6 (IL-6), and tumor necrosis factor alpha (TNF-α) elicit a sickness behavior, which is characterized by social withdrawal, lethargy, and loss of thirst and appetite (4).

An injection with LPS can be used to model aspects of sickness. Recent studies in pigs indicate changes in behavior and brain physiology after an injection of LPS (5, 6). At 2 days post-injection, the LPS-injected pigs exhibited a shift in social motivation and performed more tail- and ear-directed behavior than saline-injected pigs (5). At 3 days post-injection, the LPS-injected pigs had higher levels of IFN-γ in their frontal cortex, a tendency toward an elevation of IL-18 in their right hippocampus, and lower levels of noradrenaline in their hypothalamus, hippocampus, and frontal cortex compared to the saline-injected pigs (6). The downregulation of central monoamines that play an important role in the regulation of mood is one of the possible pathways through which cytokines can influence behavior (7, 8).

Another possible pathway for the cytokines to influence behavior is through the induction of the enzyme indolamine 2,3-deoxygenase (IDO) (9) by IFN-γ and TNF-α. IDO metabolizes tryptophan (TRP) to kynurenine (KYN), which is further metabolized into different neuroactive components. IDO is a critical molecular mediator of inflammation-induced depressive-like behavior. In rodents, it has been shown that a blockade of IDO activity prevents the development of depressive-like behavior, whereas administration of KYN induces a depressive-like behavior (10). A relationship between depressive symptoms and peripheral blood levels of TRP and KYN during IFN-α treatment has been reported in humans (11).

Modulation of the immune response by drugs is of major interest in human and veterinary medicine. Nonsteroidal anti-inflammatory drugs (NSAIDs) are candidate substances for blocking the effect of immune activation on behavior. This drug class targets cyclooxygenases (COX) and reduces pain and decreases fever and inflammation through the inhibition of prostaglandin synthesis (12). Additionally, some NSAIDs are able to alter the expression of NFkappaB (13), a transcription factor that is activated by the binding of LPS to TLR-4 (14), and thereby reduce subsequent cytokine expression. Ketoprofen, which inhibits the COX-1 and COX-2 enzymes (15), is a commonly used NSAID in veterinary medicine and has been repeatedly established as a major anti-inflammatory drug in pigs (16, 17). An effect on NFkappaB has not been shown, but other effects apart from COX inhibition and PGE2 reduction cannot be ruled out (18).

Most studies of LPS effects focus on a relatively short time period (24 h) and rarely describe social behavior in detail (9, 19, 20). In recent LPS studies on social behavior and brain physiology, pigs were either housed in groups of three (5) or singly (6). Thus, the complexity of social interactions that could be studied was limited. The pig is a gregarious species, and investigation of their social behavior in larger groups would increase the understanding of how immune activation can influence social dynamics and the likelihood of damaging behaviors. These behaviors are a major welfare challenge in commercial pig husbandry, and a positive correlation with poor health has been reported (21, 22). Therefore, we tested the effect of a controlled immune activation with LPS on brain physiology and social behavior in pigs housed in groups of six. In addition, we tested the effect of ketoprofen intervention on the physiological and behavioral effects of LPS.

We hypothesized that a controlled immune activation would lead to changes in behavior thought to increase the risk of tail biting or ear biting and that these changes would be associated with changes in neurotransmitter and neurotransmitter precursor levels. We predicted that LPS would first decrease activity and then lead to a more manipulative behavior toward pen mates. Because downregulation of central monoamines and plasma TRP depletion are possible pathways through which cytokines can influence behavior, we predicted that LPS would decrease the levels of noradrenaline, serotonin, and dopamine and increase the cytokine levels in the CNS compared to healthy controls. We also predicted an increase in peripheral and central kynurenine and a corresponding decrease in tryptophan. NSAID treatment was predicted to attenuate the effects of immune activation on neurotransmitter and cytokine levels and on behavior.

## Materials and Methods

### Animals and Husbandry

The national animal research authority approved the experiment (FOTS ID 15232). The experiment took place between March 23 and May 15, 2018 at the Livestock Production Research Center of the Norwegian University of Life Sciences (NMBU), Ås Campus. Seventy-eight pigs aged between 11 and 12 weeks were used in the study (52 females and 26 castrated males). Only the females were included in the treatment or control groups, and their average weight on the day before treatment was 33.9 ± 9.7 kg. The pigs were group-housed by litter with six pigs per pen (four females and two males), resulting in 13 pens in total, at a stocking density of 1.3 m^2^ per pig. The four female pigs in each pen were randomly allocated to one of four treatments each so that all treatments were represented in all pens, resulting in 13 pigs per treatment. The male pigs were companion pigs used to increase the stocking density and group size. The pigs in each pen had visual and limited tactile contact with pigs from an adjoining pen. The lying area (2.4 m × 1.6 m) had a concrete floor; the rest of the pen (2.4 m × 1.6 m) was fully slatted. The pigs were fed pellets *ad libitum*, with an animal-to-feeding-place ratio of 3:1 and a diet composition of 15.5% protein, 9.0 g calcium, and 1.9 g sodium (IDEAL S Die Ekstra, produced by Norgesfôr, Mysen, Norway). Each pen had three nipple drinkers. The staff provided two handfuls of wood shavings per pen, spread on the lying area, and two handfuls of grass silage twice per day (one handful in a rack and one handful on the floor of the lying area, respectively). The slatted area in each pen was equipped with a water sprinkler, which turned on every 10 min for 20 s. Lights were on between 6 a.m. and 10 p.m. During the night, the room was dimly lit by night lights. Average ambient temperature in the unit was set to 20°C.

### Experimental Design and Sampling Procedures

The four treatments consisted of four substance combinations: saline–saline (SS), saline–LPS (SL), ketoprofen–saline (KS), and ketoprofen–LPS (KL). The first substance was administered intramuscularly (i.m.) in the neck and the second substance intravenously (i.v.) through an ear vein catheter 60 ± 14 min afterwards on average. The pigs were humanely killed at 72 h after the intravenous injection using a mixture of tiletamine and zolazepam (Zoletil Forte vet.), medetomidine (Domitor vet.), and butorphanol (Dolorex vet.), followed by pentobarbital (Eutasol vet.). Details on drug dosages, suppliers, and routes of administration are given in Table 1. Each pen was equipped with one camera placed centrally on the ceiling above the pen. The pigs were individually marked on the back, and video recordings of behavior ran continuously throughout the experiment using the Media Recorder system from Noldus (Wageningen, The Netherlands). Saliva samples were taken at baseline (between 08:30 and 10:45) and at 4, 24, 48, and 72 h after the intravenous injection. Each pig chewed on a dental cotton pad suspended on a dental cord until it was moistened [modified from Munsterhjelm et al. (23)]. Each pad was fixed within the upper part of a 10-ml sampling tube and centrifuged for 5 min at 1,000 × g to extract the saliva. Saliva was pipetted to 2-ml Eppendorf tubes and stored on dry ice until it was moved to a freezer set at −80°C at the end of a sampling day. Blood samples were taken through a 1-ml syringe from a temporarily placed ear vein catheter (20 G, Becton Dickinson Infusion Therapy AB, Sweden) at baseline (between 10:00 and 14:00) and 72 h after the i.v. injection of LPS or saline. The catheter was removed directly after the procedure. The blood was immediately transferred to 2-ml EDTA tubes and centrifuged for 10 min at 1,000 × g. The plasma was pipetted to 2-ml Eppendorf tubes and stored on dry ice until it was moved to a freezer set at −80°C at the end of a sampling day. Brain samples were taken 10 ± 2 min on average after euthanasia (i.e., 72 h after i.v. injection of LPS or saline). The head was removed from the body, and the skull was opened using a wood saw designed to cut when pulled toward the operator and chisel. The brain was removed, and the frontal cortex (left and right), hippocampus (left and right), hypothalamus (left and right), and brain stem (left and right) were collected. The frontal cortex was sampled by placing a transverse section ~2 cm caudal to the apex of the frontal lobe. The hippocampus was obtained by blunt dissection after having cut through the corpus callosum to separate the left and the right hemispheres down to the level of the thalamus. The hypothalamus was collected by using the optic chiasm and the corpus mammilare (included in the sample) as anatomical reference points. Underlying tissue was included by placing two section lines at 45° to the imaginary line between the optic chiasm and the corpus mammilare so that the tissue block resembled a triangle. The brain stem was sampled to include both the locus coerulus and the raphe nuclei. Rostrally, the brain stem sample was delimited by a diagonal cut placed from the end of the hypothalamus sample ventrally to just caudal to the inferior colliculi (not included in the sample). Caudally, the brain stem sample was cut approximately at the caudal end of the pons. The samples were snap-frozen in isopentane on dry ice and moved to a freezer set at −80°C at the end of a sampling day.

**Table 1:** Overview of substances used for each procedure, with dose, and route of administration indicated.

**Substance**	**Generic name, concentration, and supplier**	**Dose per kilogram body weight and route of administration**	**Procedure**
Butorphanol	Dolorex MSD Animal Health (10 mg ml^−1^), The Netherlands	0.18 mg kg^−1^ IV	Anesthesia prior to euthanasia
Ketoprofen	Romefen vet (100 mg ml^−1^)	6 mg kg^−1^ IM	Treatment
	Ceva Santé Animale, France		
Lidocaine	Xylokain 5% ointment	Topical	Topical application prior to catheterization of the ear vein
	Aspen Pharma Trading Ltd., Irland		
Lipopolysaccharide	Serotype 0111: B4 of *Escherichia coli* dissolved in 0.9% sterile saline to a concentration of 100 μg ml^−1^	1.2 μg kg^−1^ IV	Treatment
	Sigma, Germany		
Medetomidine	Domitor vet. (1 mg ml^−1^)	0.06 mg kg^−1^ IV	Anesthesia prior to euthanasia
	Orion Pharma International, Finland		
Sodium chloride	9 mg ml^−1^	IV	Control treatment
Sodium pentobarbital	Euthasol vet. (400 mg ml^−1^)	140 mg kg^−1^ IV	Euthanasia
	Le Vet, The Netherlands		
Tiletamine	Zoletil Forte vet. (50 mg ml^−1^)	2.84 mg kg^−1^ IV	Anesthesia prior to euthanasia
	Virbac, Norway		
Zolazepam	Zoletil Forte vet. (50 mg ml^−1^)	2.84 mg kg^−1^ IV	Anesthesia prior to euthanasia
	Virbac, Norway		

### Video Analysis

All behavioral recordings were analyzed using the Observer XT 14.1 from Noldus (Wageningen, The Netherlands).

#### Behavioral Signs of Sickness

Behavioral signs of sickness were observed on the day of injection, referred to as DAY1, by one observer (JV) who was blinded to the treatment. Instantaneous scan sampling was performed every 5 min for 6 h after the injection of the last pig in the pen for all treatment and control pigs. All 13 pens were included in the analysis. The ethogram used is displayed in Table 2.

**Table 2:** Ethogram for behavioral signs of sickness.

**Behavior**	**Description**
Lying lateral	Lying on the flank with the head resting on the ground and not moving; body (parts) may make rapid, sudden, short-lasting movements
Lying sternal	Lying on the sternum with the head resting on the ground; body (parts) may make rapid, sudden, short-lasting movements
Lying alert	Lying (on flank or sternum) with the head up
Feeding	Snout in feeder
Active	Any active behaviors in standing position except feeding, including moving, exploration, social behavior, drinking, elimination, and comfort behavior
Interruption	Person is in the pen; scan not included in data analysis

#### Social Behavior

Continuous observation of performers and receivers of social behavior at certain intervals during the day was performed by one observer (CV) who was blinded to the treatment and day of experiment. The sampling scheme for the baseline day (1 day before injection), referred to as DAY0, was four 15-min intervals in the morning (08:00–08:15, 08:30–08:45, 09:00–09:15, and 09:30–09:45) and six 15-min intervals in the afternoon (14:00–14:15, 14:30–14:45, 15:00–15:15, 15:30–15:45, 16:00–16:15, and 16:30–16:45). The same sampling scheme was applied on the day after the injection, referred to as DAY2, and on the second day after the injection, referred to as DAY3. The day of injection itself (DAY1) was not of interest for observation as social behavior was interrupted due to handling for injections and saliva and blood sampling. The sampling scheme resulted in 150 min of continuous observation per pen for each of the days DAY0, DAY2, and DAY3. If a person entered the pen within a 15-min observation interval (e.g., due to saliva sampling, silage feeding, cleaning of pen, marking and weighing of pigs), the observation was postponed until the person had left the pen, and the interval was extended to obtain 15 min of observation. Due to the inadequate quality of the video material from one pen (which was too brightly lit to identify back markings), only 12 out of 13 pens were included in the analysis. The ethogram for social behavior is displayed in Table 3.

**Table 3:** Ethogram for social behavior.

**Behavior**	**Description**
Tail manipulation	Touching the tail of another pig with the snout, including taking the tail into the mouth
Ear manipulation	Touching the ear of another pig with the snout, including taking the ear into the mouth
Flank nosing	Touching the flank region (upper part of the lateral side of the body from the beginning of the shoulder until the end of the body, except the tail) of another pig with the snout
Belly nosing	Repetitive up-and-down movements on the abdomen of another pig that is lying or standing
Manipulation of other body parts	Touching body parts of another pig with the snout except for tail, ear, belly, and flank region (e.g., head, legs, back), including taking the body parts into the mouth
Fighting	Biting, hitting, and knocking of another pig with the head; includes chasing performed immediately after biting, hitting, and knocking; includes parallel pressing after a knock, hit, or bite. The pig that initiates the fight is the performer; the pig that is being attacked is the recipient
Displacement	Pushing away another pig without fighting (as defined above); results in the active movement of the recipient and getting access to a resource (e.g., silage, lying space, drinker) for the performer

### Lab Procedures and Measurements

#### Cortisol

Cortisol in saliva was measured using an enzyme immunoassay kit (DetectX®, Catalog Number K0033-H5W, Arbor Assays, MI, USA). The saliva samples were thawed and centrifuged at 4°C at 2,500 × g for 20 min. The kit reagents were prepared according to the manufacturer's protocol. In total, 30 μl of each sample was transferred to Eppendorf tubes, diluted by adding 120 μl of assay buffer (1:4 dilution), and vortexed. All samples were measured within 2 h of preparation. All standards, nonspecific binding wells, blanks, and samples were run in duplicate. Treatments were distributed randomly over the plates. In total, 50 μl of samples, quality controls (high/low), or standards were pipetted into appropriate wells (96-well plate). Each well then received 25 μl of DetectX® cortisol conjugate, followed by 25 μl of DetectX® cortisol antibody (except for low-quality controls), using a repeater pipette. After incubating the plate on a shaker at room temperature for 1 h, the plate was aspired, and each well was washed four times with 300 μl of wash buffer. Then, 100 μl of 3,3′,5,5′-tetramethylbenzidine solution was added to each well, and the plate was incubated for 30 min at room temperature. After this time, 50 μl of stop solution (1 M hydrochloric acid) was added before the optical density of each well was read with the Sunrise Absorbance Reader (Tecan Austria GmbH, Grödig/Salzburg, Austria) at 450 nm using the Magellan 6.4 software. Mean coefficient of variation varied between 4.69 and 7.63%. Sensitivity was determined as 27.6 pg ml^−1^, and limit of detection was determined as 45.4 pg ml^−1^ according to the manufacturer.

#### Homogenization of Brain Tissue

The frozen brain tissue blocks were mechanically homogenized using a pestle and a mortar that was placed on dry ice and filled repeatedly with liquid nitrogen to keep the sample frozen. The pulverized brain tissue was transferred into 2-ml round-bottomed Eppendorf tubes and weighed. Ten tubes with tissue from the frontal cortex (five left and five right), hippocampus (five left and five right), and brain stem (five left and five right), respectively, and six tubes with tissue from the hypothalamus (three left and three right) were collected and stored at −80°C until analysis.

#### Cytokines

For cytokine analysis, a 500-mM phenylmethylsulfonyl fluoride (PMSF) solution was prepared by dissolving 0.436 g PMSF in 5 ml dimethylsulfoxide. PMSF solution (80 μl) was added to a lysing solution that contained cell lysis buffer (19.8 ml) and factor 1 (80 μl) and factor 2 (40 μl) of a cell lysis kit (Bio-Rad, #171-304011). In total, 500 μl of the prepared lysis solution and a 5-mm tungsten bead were added to each sample tube (one tube per brain region). The samples were mechanically homogenized at room temperature for 4 min at 20 Hz using the TissueLyser 2 (Cat. No 85300, Qiagen). The homogenate was centrifuged at 4,500 × g for 20 min at 4°C (Heraeus Multifuge 3SR+ Centrifuge, Thermo Fisher Scientific, MA, USA). The supernatant was transferred to new Eppendorf tubes and stored at −20°C.

Cytokines in brain tissue were measured by a Milliplex MAP Porcine Cytokine and Chemokine Magnetic Bead Panel Immunology Multiplex Assay, including the cytokines interferon gamma (IFN-γ), tumor necrosis factor alpha (TNF-α), and interleukin 18 (IL-18) (PCYTMAG-23K; Merck, Norway). The rationale for including these cytokines was based on previous findings (6). Treatments were distributed randomly over the plates. The supernatant obtained from the homogenization of brain tissue was thawed for 10 min at room temperature, centrifuged at 4°C at 4,500 × g for 10 min and transferred to new Eppendorf tubes. The kit reagents were prepared according to the manufacturer's protocol. The thawed supernatant (50 μl) was transferred into new Eppendorf tubes and diluted by adding 49 μl assay buffer and 1 μl bovine serum albumin solution (20 %, Sigma-Aldrich, USA). In total, 25 μl of each standard or control was added to the appropriate wells using assay buffer for the 0-ng-ml^−1^ standard (background). In total, 25 μl of serum matrix solution was added to the background, standards, and control wells. In total, 25 μl of assay buffer and 25 μl of sample (neat) were transferred to the appropriate sample wells. In total, 25 μl of the mixed beads were transferred to each well, and the plate was incubated on a plate shaker at 2–6°C overnight. The well contents were removed after resting the plate on a hand-held magnet, and each well was washed three times with 200 μl of wash buffer. The plate was incubated for 2 h with 50 μl detection antibodies per well at room temperature. This step was followed by 30 min of incubation with 50 μl streptavidin–phycoerythrin and another washing step. The well content was suspended in sheath fluid, and the plate was run with the Luminex100 (Bio-Rad, Hercules, CA, USA) using the BioPlex Manager 6.0 software (Bio-Rad, Hercules, CA, USA). Sample wells with a bead count <20 were excluded from further analysis. For TNF-α, ca. 50% of the analyzed samples was below the lower limit of detection (LOD), and the calculated cytokine concentrations were consequently censored by the analysis software. Therefore, we used fluorescence intensity (FI) values for further analysis [see Nordgreen et al. (6) for further explanation]. Mean coefficient variation was 9.97% for IFN-γ (with all wells included, the range was 0–62.51), 9.50 for TNF-α (with all wells included, the range was 0–96.17), and 12.30 for IL-18 (with all wells included, the range was 0–78.0). The minimum detectable concentration was 0.042 ng ml^−1^ for IFN-γ, 0.006 for TNF-α, and 0.012 for IL-18. In order to correct for sample weight, the values for observed concentration (divided by two) and the values for fluorescence intensity were divided by the sample weight.

#### Monoamines, Tryptophan, and Kynurenine

A sensitive and selective high-performance liquid chromatography–electrospray ionization–tandem mass spectrometry (HPLC–ESI–MS/MS) method for quantification of several neurotransmitters, amino acids, and their metabolites was developed by co-author DD. These included TRP and its metabolite KYN, the three neurotransmitters dopamine (DA), serotonin (5-HT), and noradrenaline (NA), and their respective metabolites 3,4-dihydroxyphenyl-acetic acid (DOPAC), homovanillic acid (HVA), 5-hydroxyindoleacetic acid (5-HIAA), and 3-methoxy-4-hydroxyphenylglycol (MHPG). The pure compounds and their respective corresponding stable isotope-labeled standards were provided from CDN Isotopes (Quebec, Canada), Sigma-Aldrich (Darmstadt, Germany), and TRC (Toronto, Canada). All chemicals were of at least HPLC-grade and supplied by VWR International (Fontenay sous Bois, France). All samples were thawed on ice, and the respective corresponding isotope-labeled internal standards were added according to the weight/volume of each sample. The brain samples (sample size: 20–500 mg) were homogenized with acetonitrile 1:5 (v/w) on ice and then centrifuged at 12,000 × g for 30 min at 4°C. The plasma samples (sample size: 15 to 50 μl) were precipitated with acetonitrile 1:5 (v/v) after addition of the internal standard mixture. After being vortexed for 30 s and centrifuged at 12,000 × g for 15 min, 50 μl of plasma samples and, respectively, 100 μl of brain samples supernatants were subsequently transferred into a new 15-ml polypropylene centrifuge tube and evaporated to dryness at 40°C under a nitrogen stream in a water bath (Zymark Turbo Vap LV, Oregon, USA) (24, 25). The dry residue was reconstituted in 100 μl dilution solution of 10% methanol/water (v/v) with 0.1% formic acid and 0.05% ascorbic acid, filtered with Spin-X centrifuge tube filter, 0.22 μm (Costar, UT, USA), and transferred to a HPLC vial with insert (Agilent, Santa Clara, CA, USA). The HPLC–ESI–MS/MS system was performed using an Agilent 1100 setup consisting of a binary pump, degasser, and autosampler with thermostat (Agilent Technologies, Santa Clara, CA, USA) coupled to an API 4000 triple–quadrupole mass spectrometer (AB Sciex, Ontario, Canada) equipped with a turbo ion spray. The temperature of the autosampler was set at 5°C. Chromatographic separation was carried out on a reversed-phase Synergy–Fusion column, 100 mm × 2.1 mm, 2.5-μm particles (Phenomenex, CA, USA), with a Fusion-RP guard column. The column running temperature was 25°C. The mobile phase consisted of 0.1 % acetic acid in water (A) and acetonitrile/methanol (50% v/v) (B). The separated compounds were detected in positive and negative electrospray ionization-multiple reaction monitoring mode using the respective [M+H]^+^ (protonated) and [M-H]^−^ (deprotonated) ions in two separate run analyses, selecting one precursor ion to two product ion transitions for each compound. The positive ion transitions used for quantification were as follows: DA (m/z 154 > 137), DA-d4 (m/z 158 > 141), 5-HT (m/z 177 > 160), 5-HT-d4 (m/z 181 > 164), NA (m/z 170 > 107), NA-d6 (m/z 176 > 111), TRP (m/z 205 > 188), 13C-TRP (m/z 206 > 189), d5-TRP (210 > 192) (used for plasma samples only), KYN (m/z 209 > 192), and KYN-d4 (m/z 213 > 196). The negative ion transitions used for quantification were as follows: DOPAC (m/z 167 > 123); DOPAC-d5 (m/z 172 > 128); HVA (m/z 181 > 137), HVA-d5 (m/z 186 > 142), 5-HIAA (m/z 190 > 146), 5-HIAA-d5 (m/z 195 > 151), MHPG (m/z 183 > 150), and MHPG-d3 (m/z 186 > 150). The software used for controlling this equipment and for acquiring and processing the data was Analyst Version 1.7 (AB Sciex, Ontario, Canada). Since the analytes are endogenous components in biological matrices, the validation parameters (detection limit, linearity, precision, accuracy, recovery, and matrix effects) (26) were determined by spiking the brain homogenate and the plasma as matrices, with the corresponding stable isotope-labeled standard analogs of each compound as surrogate standard (27). Standard stock solutions were prepared in methanol at 1 mg ml^−1^, except kynurenine, which was dissolved in dimethyl sulfoxide. Working solutions were prepared in dilution solution [10% methanol/water (v/v) with 0.1% formic acid and 0.05% ascorbic acid]. All the solutions were stored at −20°C. The calibration standards were prepared in dilution solution as surrogate matrix based on the correction factors calculated for each compound related to their respective matrix effects and recovery values (27). The linear ranges were as follows: 0–500 ng ml^−1^ for DA, 0–3,000 ng ml^−1^ for NA, 0–2,000 ng ml^−1^ for 5-HT, 0–250 ng ml^−1^ for KYN, 0–5,000 ng ml^−1^ for TRP, 0–250 ng ml^−1^ for DOPAC, 0–200 ng ml^−1^ for MHPG, 0–500 ng ml^−1^ for HVA, and 0–350 ng ml^−1^ for 5-HIAA, respectively, corresponding to 50 mg brain tissue scales, and 0–500 ng ml^−1^ for KYN and 0–15,000 ng ml^−1^ for TRP corresponding to 50 μl plasma. The calibration curves were constructed based on the peak area ratio of the analytes to internal standards vs. the nominal concentration ratio (analyte to internal standard). The concentration in each sample was calculated using the peak area ratio and linear regression analysis. The response for each compound was linear and gave a correlation coefficient of *r*^2^ ≥ 0.99. LOD was based on 3 × signal-to-noise ratio, and a lower limit of quantification (LLOQ) was determined as the lowest concentration validated. LLOQs ranged between 1 and 10 ng ml^−1^ for all the compounds. Two quality control samples were used for each run of analysis and prepared in a real brain homogenate by spiking with a known concentration of each analyte in order to evaluate the inter-assay precision and accuracy of the method (94.41 ± 5.88–112.58 ± 5.73%). The extraction recoveries were between 50 and 95% for all analytes, except 5-HIAA with 20%. The use of the stable isotope-labeled internal standard is one of the approaches to correct for matrix effects and improve the accuracy and precision of the analytical method.

### Statistical Analysis

The significance level for all analysis was set at *p* < 0.05. Standard deviations were used. Residuals were checked for normality and homogeneity of variance by visual inspection of plots. If they did not fulfill either of these criteria, raw data were transformed. Main effects are not presented when the interaction was in focus to answer the research question. *A priori* planned contrasts were used after running the main models as we had predefined the hypotheses to test [for further explanation, see Doncaster and Davey (28)].

#### Behavioral Data

Behavioral data were analyzed using mixed models in JMP Pro 14.3.0 (SAS, NC, USA). The removal of outliers did not improve the respective model fit (Akaike information criterion, AIC); thus, they were included in further analysis. For behavioral signs of sickness, the frequency of the respective behavioral pattern (lying lateral/sternal/alert, feeding, being active) was used as dependent variable, and the treatment (SS, SL, KS, KL), the hour after the injection (1, 2, 3, 4, 5, and 6 h), and the interaction of both were used as independent fixed effects. Pig nested in treatment was included as a random variable in all models. For planned comparisons between treatment groups at the different time points, Student's *t*-test was used after running the main model. We compared SL with SS to elucidate the effect of LPS on behavioral signs of sickness. In addition, the comparison of SL and KL should answer the question whether ketoprofen alleviates the effects of LPS. Furthermore, it was relevant to compare SS with KS in order to see whether ketoprofen has an effect even in pigs that are not sick.

For social behavior, the frequency and duration of each behavioral pattern performed and received (manipulation of tail/ear/other body parts, nosing of belly/flank or fighting and displacement) were skewed to the right, the model residuals did not confirm with the criteria listed in “Section Statistical analysis,” and the raw data were therefore square root-transformed. The square root of frequency and duration of the respective behavior was used as dependent variable and treatment (SS, SL, KS, and KL) and day in relation to LPS injection (DAY0, DAY2, and DAY3) as fixed effect. The interaction between treatment and day was included in the model as the factor of most interest for testing our hypothesis of an effect of LPS and ketoprofen on the dependent variables. Pig nested in treatment was included as a random variable in all models. For planned comparisons, Student's *t*-test was used. In a first step, we compared SL with SS, SL with KL, and SS with KS within each day. If any of these pairwise comparisons were significant, we compared within-group differences between baseline and the day at which the significant treatment effect was found.

#### Physiological Data

Physiological data (cortisol, cytokines, tryptophan, kynurenine, monoamines) were analyzed using mixed models in JMP Pro 14.3.0 (SAS, NC, and USA). The removal of outliers did not improve the respective model fit (AIC); thus, they were included in further analysis.

For salivary cortisol, the square root of the observed concentration (ng ml^−1^) was used as dependent variable and treatment (SS, SL, KS, and KL) and sampling time point in relation to i.v. injection (T0, T4, T24, T48, and T72) as fixed independent variables. The interaction between treatment and sampling time point was included in the model as the factor of most interest for testing our hypothesis of an effect of LPS and ketoprofen on the dependent variable. Pig nested in treatment was included as a random variable in the model. For planned comparisons of group means at the same time points, Student's *t*-tests was used on the LSmeans after running the full mixed model. We compared SL with SS, SL with KL, and SS with KS.

For brain cytokines, the square root of the observed concentration (ng g^−1^) of IFN-γ and IL-18 and the FI of TNF-α were used as dependent variables. The treatment (SS, SL, KS, and KL) and the hemisphere (left, right) were fixed effects. The time span (TIME) between death of the respective pig until the last brain sample was collected and frozen was included as a covariate in all models. In case of nonsignificance, TIME was removed from the final model. Pig nested in treatment was included as a random variable in all models. For planned comparisons of treatments, Student's *t*-test was used. We compared SL with SS, SL with KL, and SS with KS. Analyses were run for each brain area separately as a difference between brain areas was not of interest.

For tryptophan and kynurenine, the observed concentration (ng ml^−1^ in plasma and ng g^−1^ in brain tissue) and the kynurenine–tryptophan ratio (calculated as KYN/TRP) were used as dependent variables. The ratios between KYN and TRP were calculated as a ratio between the mol mg^−1^ or mol ml^−1^ of each of the analytes included in the ratio. For modeling the plasma concentrations of the respective analyte, treatment (SS, SL, KS, and KL) and sampling time point (T0, T72) were used as independent variables. The interaction between treatment and sampling time point was included in the model as the factor of most interest for testing our hypothesis of an effect of LPS and ketoprofen on the dependent variable. For modeling brain concentrations, treatment (SS, SL, KS, and KL) and hemisphere (left, right) were used as independent variables. TIME from euthanasia until the brain tissue was frozen was included as covariate in all models and removed in case of non-significance. Pig nested in treatment was included as a random variable in all models. For planned comparisons of treatments, Student's *t*-test was used. We compared SL with SS, SL with KL, and SS with KS. Analyses were run for each brain area separately as a difference between brain areas was not of interest. For correlations between the concentrations in plasma and brain tissue at 72 h after the injection, Spearman rank coefficient was used. In total, 37 pigs were included in this analysis.

For brain monoamines, the observed concentration (ng g^−1^) of the respective analyte (DA, NA, 5-HT) as well as the ratio between metabolite(s) and mother substance were used as dependent variables. Dopamine turnover [calculated as (DOPAC + HVA/DA)], serotonin turnover (calculated as 5-HIAA/5-HT), and noradrenaline turnover (calculated as MHPG/NA) were calculated as an index for the activity of the dopaminergic, serotonergic, and noradrenergic systems. High levels indicate a higher activity in the respective system. Ratios were calculated based on moles per milligram of the respective analytes. Treatment (SS, SL, KS, and KL) and hemisphere (left, right) were used as independent variables. TIME was included as covariate in all models and removed in case of nonsignificance. Pig nested in treatment was included as a random variable in all models. For planned comparisons of treatments, Student's *t*-test was used. We compared SL with SS, SL with KL, and SS with KS. Analyses were run for each brain area separately as a difference between brain areas was not of interest.

## Results

### Behavioral Signs of Sickness

**Figure 1:**
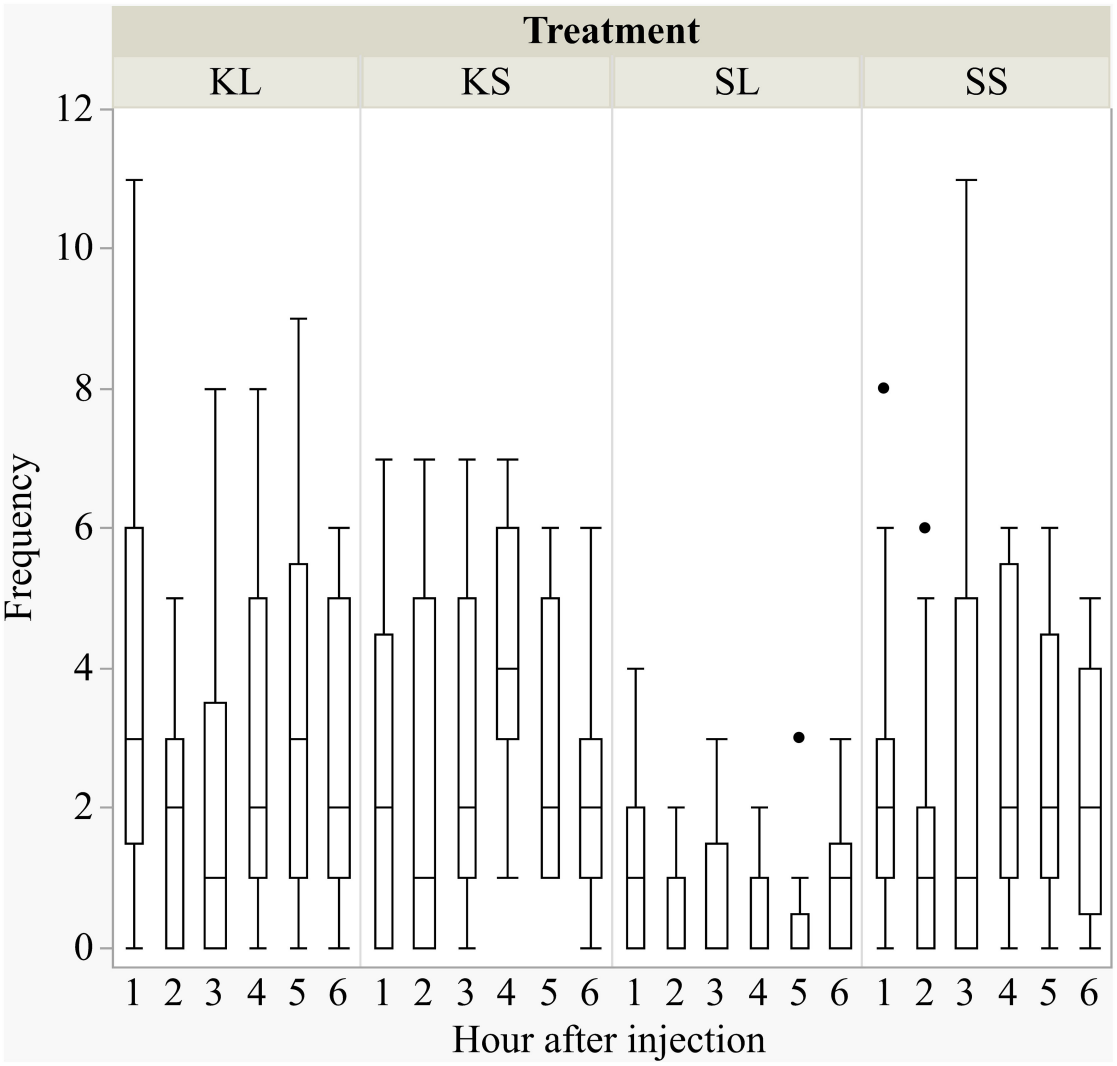
Frequencies (per hour) of active behavior after injecting the pigs with ketoprofen–lipopolysaccharide (LPS), ketoprofen–saline, saline–LPS, and saline–saline. Round dots indicate outliers. *N* = 13 pigs per treatment.

LPS had an effect on general activity, feeding, and lying behavior, and ketoprofen attenuated these effects (Figure 1). LPS-injected pigs (SL) were significantly less active [*F*(treatment × hour)_(15,240)_ = 0.98, *p* = 0.47] than saline-injected pigs (SS) at 3 h (planned comparison: *p* = 0.017), 4 h (*p* = 0.002), and 5 h (*p* = 0.006) after the challenge. Feeding was depressed at the same time points [*F*_(15,240)_ = 1.45, *p* = 0.13, planned comparison: 3 h: *p* = 0.01, 4 h: *p* = 0.004, 5 h: *p* = 0.002]. SL pigs compared to SS pigs lay more frequently with their heads up [*F*_(15,240)_ = 3.01, *p* = 0.0002] at 2 h (*p* = 0.009) and lay more sternally [*F*_(15,240)_ = 1.01, *p* = 0.44) at 4 h (*p* = 0.017) and 5 h (*p* = 0.004). LPS-injected pigs that received ketoprofen (KL) were significantly more active than untreated, LPS-injected pigs (SL) at 1 h (planned comparison: *p* = 0.0025), 4 h (*p* = 0.0025), 5 h (*p* = 0.0003), and 6 h (*p* = 0.013) after the challenge. This corresponds to the finding that SL pigs showed more sternal recumbency compared to KL pigs at 1 h (*p* = 0.021) and 5 h (*p* = 0.007) and were lying more on the side [*F*_(15,240)_ = 1.55, *p* = 0.09) at 3 h (*p* = 0.034), 5 h (*p* = 0.0018), and 6 h (*p* = 0.039) after the challenge.

### Social Behavior

**Figure 2:**
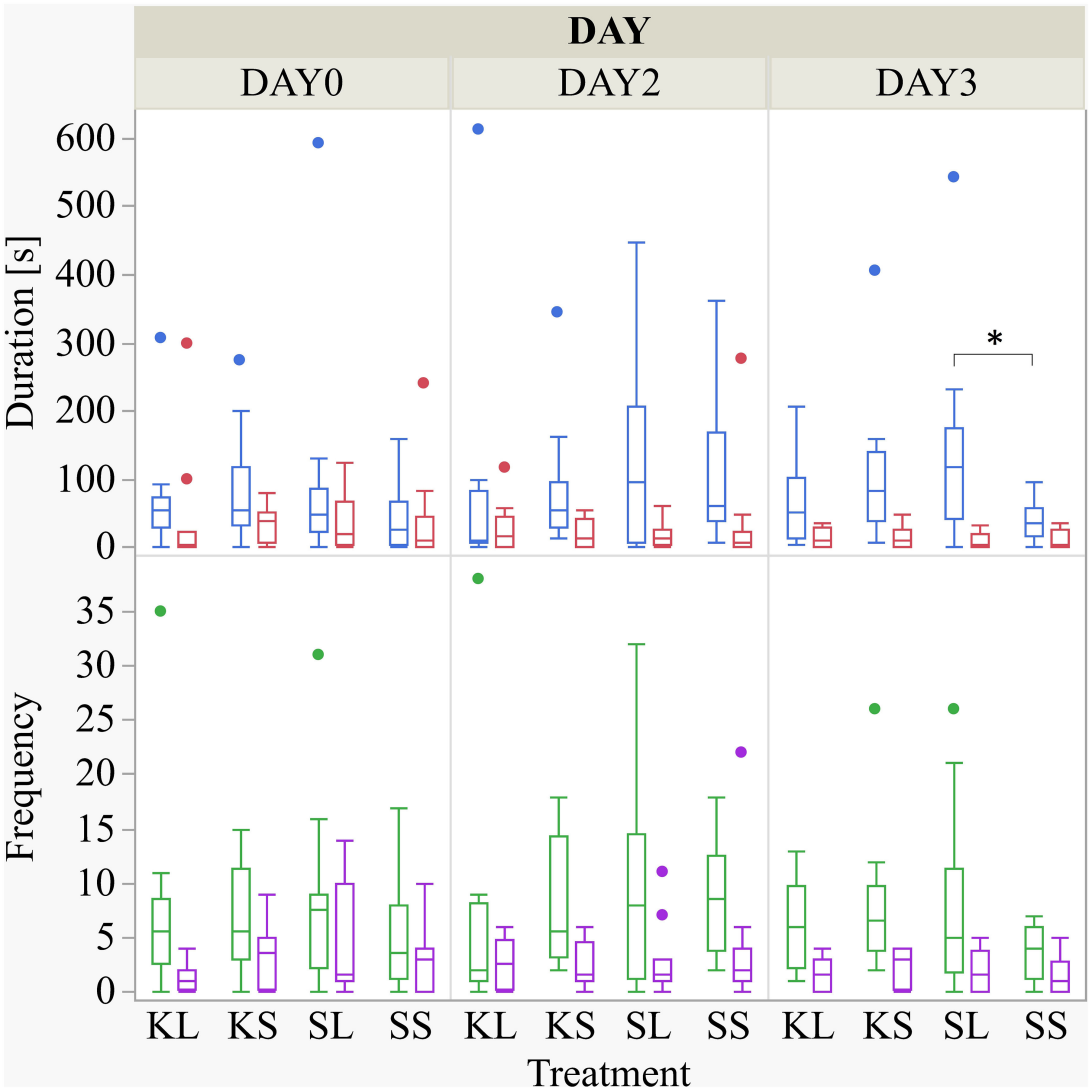
Duration (s) and frequency of performed ear manipulation (blue and green boxplot, respectively) and tail manipulation (red and lilac) at baseline (DAY0) and at the first (DAY2) and second day (DAY3) after injecting the pigs with ketoprofen–lipopolysaccharide (LPS), ketoprofen–saline, saline–LPS, and saline–saline. Round dots indicate outliers. Significant differences of planned comparisons between treatments (*p* < 0.05) are marked with an asterisk.

The frequency and duration of all performed and received behaviors observed are displayed in the Supplementary Table A. There was a considerable variation within each group for the duration of the experiment. LPS had an effect on the duration of performed ear manipulation [*F*(treatment × day)_(6,88)_ = 1.49, *p* = 0.19] but not on any other behavioral pattern observed (Figure 2). The LPS-injected pigs were manipulating the ears of their pen mates significantly longer (median = 118.82 s; min = 0 | max = 542.36 s) than the saline-injected pigs (37.38; 0 | 97.72) 2 days after the injection (planned comparison: *p* = 0.022). Ketoprofen had no effect on social behavior, neither in LPS-injected pigs nor in saline-injected controls. All results of the analysis of variance are displayed in the Supplementary Table B.

### Cortisol

**Figure 3:**
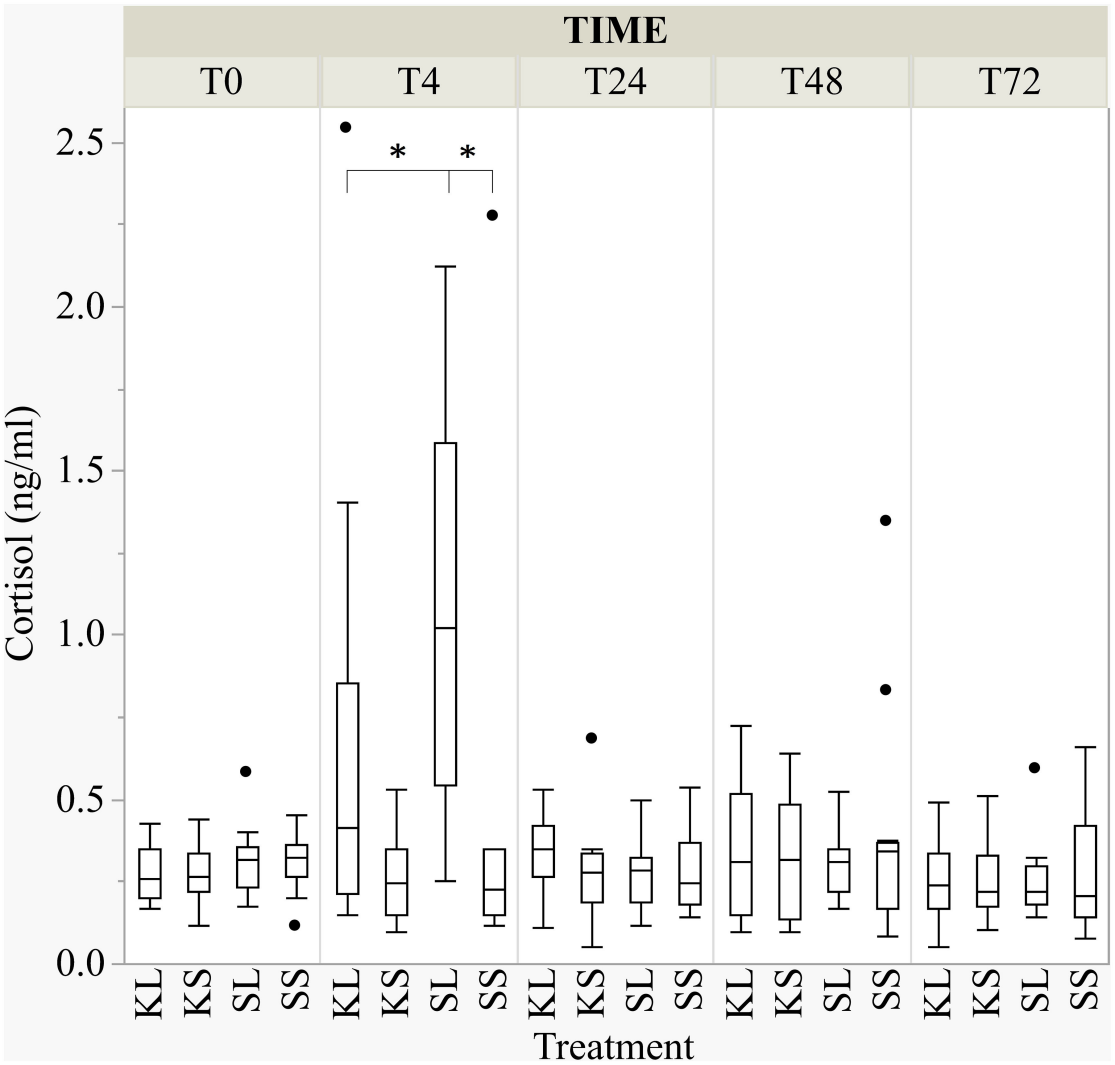
The concentration of salivary cortisol (ng ml^−1^) at baseline and at 4, 24, 48, and 72 h after injecting the pigs with ketoprofen–lipopolysaccharide (LPS), ketoprofen–saline, saline–LPS, and saline–saline. Round dots indicate outliers. Significant differences of planned comparisons between treatments (*p* < 0.05) are marked with an asterisk.

LPS activated the hypothalamic–pituitary–adrenal (HPA) axis and ketoprofen reduced this effect [*F*(treatment × time point)_(12,182.8)_ = 4.65, *p* <0.001; Figure 3]. At 4 h after the injection, salivary cortisol was significantly higher in SL pigs (mean ± SD = 1.07 ± 0.59 ng ml^−1^) compared to KL pigs (0.64 ± 0.68 ng ml^−1^; planned comparison: *p* < 0.001) and SS pigs (0.42 ± 0.62 ng ml^−1^; *p* < 0.001).

### Cytokines

The descriptive results of all cytokine concentrations according to brain region are displayed in Table 4. LPS had no observed effect on the measured brain cytokines in the regions examined at 72 h after the injection. The measured concentration of IFN-γ did not differ between treatments and hemispheres in the frontal cortex (*n* = 100 samples), hippocampus (*n* = 100), hypothalamus (*n* = 97), and brain stem (*n* = 104). A hemisphere effect on TNF-α FI for the brain stem (*n* = 104) was seen [*F*(hemisphere)_(1,51)_ = 5.51, *p* = 0.02]. The TNF-α FI levels were significantly higher in the right hemisphere (mean ± SD = 0.77 ± 0.26) compared to the left hemisphere (0.66 ± 0.25), although no treatment difference was observed. Neither treatment nor hemisphere had an effect on TNF-α FI levels in the frontal cortex (*n* = 100), hippocampus (*n* = 100), and hypothalamus (*n* = 98). Ketoprofen had an effect on IL-18 concentration in the brain stem (*n* = 104), [*F*(treatment)_(3,48)_ = 2.13, *p* = 0.11]. SS pigs had higher concentrations (0.04 ± 0.01 ng g^−1^) compared to KS pigs (0.03 ± 0.01 ng g^−1^, planned comparison: *p* = 0.018). The concentration of IL-18 did not differ between treatments and hemispheres in the frontal cortex (*n* = 100), hippocampus (*n* = 100), and hypothalamus (*n* = 98). TIME had a significant effect on IFN-γ in the hypothalamus and brain stem and on TNF-α FI levels in the hypothalamus as reflected by decreasing concentrations with increasing time at collection post-mortem. All results of the analysis of variance (ANOVA) of IFN-γ, TNF-α, and IL-18 for treatment and hemisphere according to brain region are displayed in the Supplementary Table C.

**Table 4:** Mean (± standard deviation) of concentrations (ng g^−1^) of interferon gamma (IFN-γ) and interleukin 18 (IL-18) and fluorescence intensity (arbitrary units) of tumor necrosis factor alpha (TNF-α) in the frontal cortex, hippocampus, hypothalamus, and brain stem of pigs at 72 h after injecting the pigs with saline–saline (SS), saline–lipopolysaccharide (LPS) [SL], ketoprofen–saline (KS), and ketoprofen–LPS (KL).

**Brain area**	**IFN-γ**				**TNF-α**				**IL-18**			
	**SS**	**SL**	**KS**	**KL**	**SS**	**SL**	**KS**	**KL**	**SS**	**SL**	**KS**	**KL**
Frontal cortex	0.53 ± 0.68	0.37 ± 0.36	0.57 ± 0.78	0.40 ± 0.50	0.84 ± 0.51	0.79 ± 0.52	0.60 ± 0.26	0.78 ± 0.50	0.16 ± 0.06	0.14 ± 0.06	0.14 ± 0.04	0.15 ± 0.06
Hippocampus	0.32 ± 0.24	0.54 ± 0.66	0.33 ± 0.21	0.34 ± 0.23	0.65 ± 0.34	1.03 ± 1.37	0.90 ± 0.69	0.60 ± 0.40	0.04 ± 0.01	0.04 ± 0.01	0.04 ± 0.01	0.05 ± 0.02
Hypothalamus	0.24 ± 0.10	0.24 ± 0.08	0.25 ± 0.08	0.26 ± 0.08	1.46 ± 0.72	1.43 ± 0.46	1.38 ± 0.49	1.44 ± 0.65	0.04 ± 0.01	0.04 ± 0.01	0.04 ± 0.01	0.04 ± 0.01
Brain stem	0.16 ± 0.05	0.15 ± 0.05	0.15 ± 0.06	0.15 ± 0.05	0.75 ± 0.22	0.67 ± 0.20	0.79 ± 0.36	0.67 ± 0.23	0.04 ± 0.01	0.03 ± 0.01	0.03 ± 0.01	0.03 ± 0.01

### Tryptophan and Kynurenine

#### Plasma

**Figure 4:**
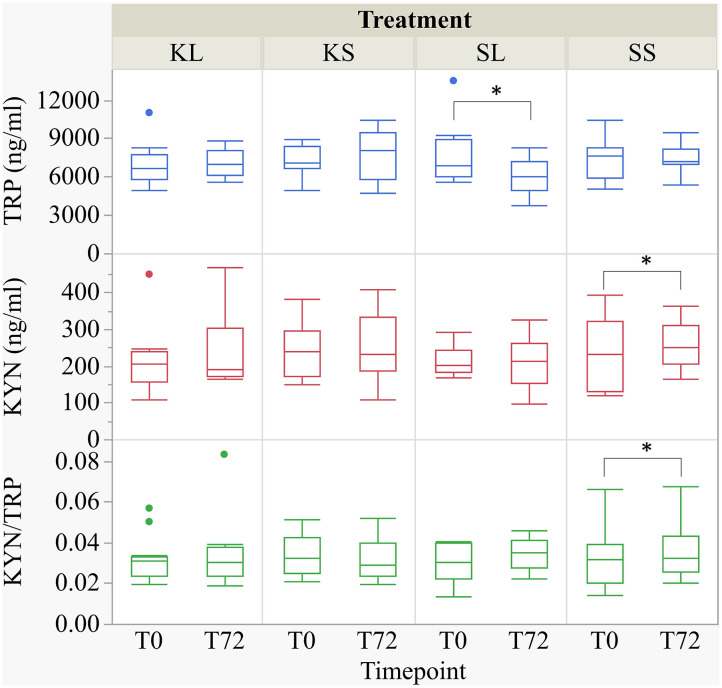
Tryptophan (ng ml^−1^), kynurenine (ng ml^−1^), and kynurenine–tryptophan ratio in pig plasma at baseline and at 72 h after the injection with ketoprofen–lipopolysaccharide (LPS), ketoprofen–saline, saline–LPS, and saline–saline. Turnover rates were calculated based on moles per milligram of the respective analyte. Round dots indicate outliers. Significant differences of planned comparisons between sampling time points (*p* < 0.05) are marked with an asterisk.

LPS had an effect on TRP concentrations in the plasma [*F*(treatment × time point)_(3,27.78)_ = 2.32, *p* = 0.097] (Figure 4). At 72 h after the injection, SL pigs had lower concentrations of TRP in their plasma (mean ± SD = 6,118.89 ± 1,451.04 ng ml^−1^) compared to baseline (7,801.11 ± 2,453.08 ng ml^−1^, planned comparison: *p* = 0.035). KYN concentrations [*F*(treatment × time point)_(3,29.37)_ = 1.64, *p* = 0.20] in SS pigs were significantly higher at 72 h after the injection (258.0 ± 65.31 ng ml^−1^) compared to baseline (227.73 ± 91.46 ng ml^−1^, *p* = 0.026). The kynurenine–tryptophan ratio [KYN/TRP, *F*(treatment × time point)_(3,27.28)_ = 2.17, *p* = 0.11] was lower at 72 h compared to baseline in SS pigs (*p* = 0.044).

The correlations between tryptophan in plasma and brain tissue were weak and positive (Spearman's rho, ρ = 0.17–0.32) but significant for the hippocampus (*p* = 0.007) and brain stem (*p* = 0.04). The correlations between kynurenine in plasma and brain tissue were positive (ρ = 0.23–0.40) and significant for the frontal cortex (*p* = 0.001), hippocampus (*p* = 0.006), and hypothalamus (*p* = 0.001).

#### Brain Tissue

**Figure 5:**
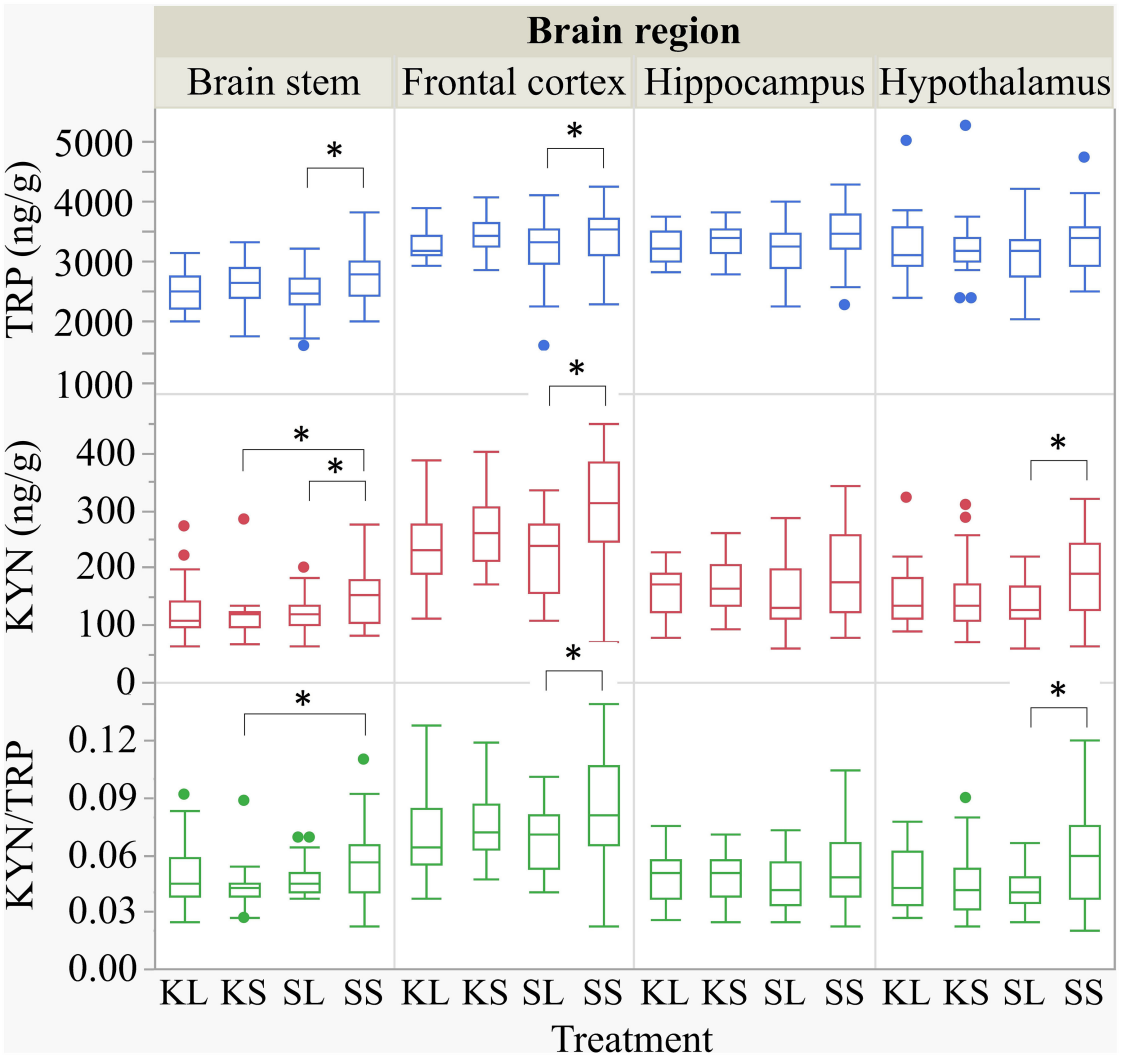
Tryptophan (ng g^−1^), kynurenine (ng g^−1^), and kynurenine–tryptophan ratio in the brain stem, frontal cortex, hippocampus, and hypothalamus of pigs at 72 h after the injection with ketoprofen–lipopolysaccharide (LPS), ketoprofen–saline, saline–LPS, and saline–saline. Turnover rates were calculated based on moles per milligram of the respective analyte. Round dots indicate outliers. Significant differences of planned comparisons between treatments (*p* < 0.05) are marked with an asterisk.

LPS injection had an effect on TRP and KYN concentrations in several brain areas measured at 72 h after administration (Figure 5). Lower concentrations of TRP were measured in the brain stem [*F*(treatment)_(3,47.94)_ = 1.90, *p* = 0.14] and the frontal cortex [*F*_(3,45.41)_ = 2.04, *p* = 0.12] of LPS-injected pigs (SL) compared to saline-injected pigs (SS, planned comparisons: *p* = 0.04, respectively) There were no differences between treatments and hemispheres in the hippocampus and the hypothalamus.

Lower concentrations of KYN were measured in the brain stem [*F*_(3,48.24)_ = 2.33, *p* = 0.09], frontal cortex [*F*_(3,47.4)_ = 4.0, *p* = 0.01], and hypothalamus [*F*_(3,47.11)_ = 2.43, *p* = 0.08] of SL pigs compared to SS pigs (planned comparison: *p* = 0.035, 0.003, 0.01). Moreover, the KYN/TRP ratio in the frontal cortex [*F*_(3,47.02)_ = 1.95, *p* = 0.13] and in the hypothalamus [*F*_(3,46.76)_ = 2.21, *p* = 0.10] was significantly lower in SL pigs compared to that in SS pigs (*p* = 0.037, 0.017).

Ketoprofen had an effect on KYN concentrations in the brain stem of saline-injected pigs. Pigs that were treated with ketoprofen (KS) had lower concentrations than the untreated controls (SS) (*p* = 0.02). That finding was reflected by a significantly lower KYN/TRP ratio in the brain stem [*F*_(3,48.25)_ = 1.81, *p* = 0.16] of KS pigs compared to that of SS pigs (*p* = 0.03). There were no differences in KYN/TRP ratio between treatments and hemispheres in the hippocampus. The covariate TIME had a significant effect on the quantification of TRP and KYN concentrations in the frontal cortex and the hippocampus as reflected by decreasing concentrations with increasing time. The same effect was seen for TRP concentrations in the hypothalamus. All results of the analysis of variance are displayed in the Supplementary Table D.

### Monoamines

**Figure 6:**
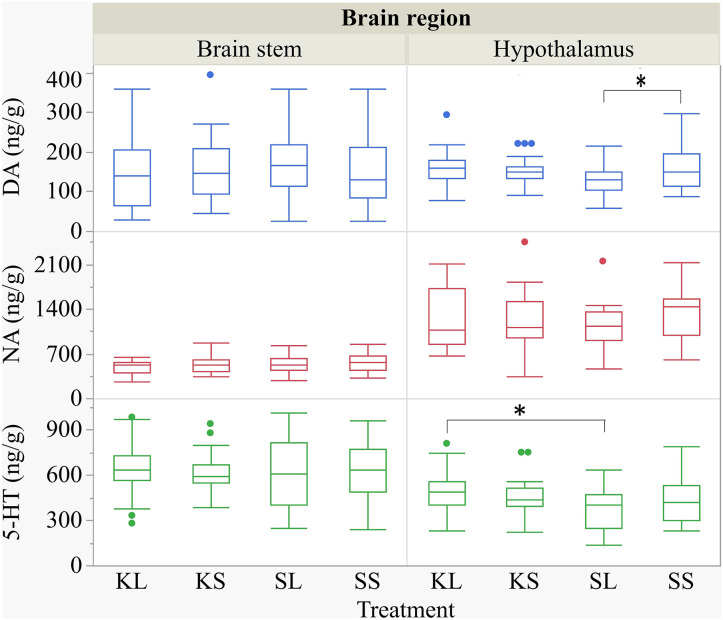
Concentration (ng g^−1^) of dopamine, noradrenaline, and serotonin in the brain stem and in the hypothalamus of pigs at 72 h after the injection with ketoprofen–lipopolysaccharide (LPS) (KL), ketoprofen–saline, saline–LPS, and saline–saline (SS). To improve the readability, two outliers were removed from the figure (one SS and one KL in the hypothalamus). Round dots indicate outliers. Significant differences of planned comparisons between treatments (*p* < 0.05) are marked with an asterisk.

The descriptive results of all monoamine concentrations according to brain region are displayed in Table 5. Highest concentrations of DA, NA, and 5-HT were found in the brain stem and the hypothalamus. LPS had an effect on DA concentrations [*F*(treatment)_(3,47.06)_ = 2.05, *p* = 0.12; Figure 6] and respective turnover rate [*F*_(3,46.16)_ = 2.28, *p* = 0.09; **Figure 8**] in the hypothalamus measured at 72 h after the challenge. SL pigs had significantly lower DA concentrations compared to SS pigs (planned comparison: *p* = 0.032). This finding is reflected by a higher DA turnover of SL pigs compared to SS pigs (*p* = 0.042). There were no differences between treatments and hemispheres in the frontal cortex, hippocampus, and brain stem.

**Table 5:** Mean (± standard deviation) of concentrations (ng g^−1^) of dopamine (DA), noradrenaline (NA), and serotonin (5-HT) as well as dopamine turnover (DOPAC + HVA/DA), noradrenaline turnover (MHPG/NA), and serotonin turnover (5-HIAA/5-HT) in the frontal cortex, hippocampus, hypothalamus, and brain stem of pigs at 72 h after the injection.

	**Frontal cortex**	**Hippocampus**	**Hypothalamus**	**Brain stem**
DA (ng g^−1^)	10.66 ± 8.45	7.03 ± 5.12	160.00 ± 73.59	157.52 ± 83.81
NA (ng g^−1^)	117.30 ± 33.15	131.61 ± 41.21	1,238.99 ± 416.00	529.42 ± 130.29
5-HT (ng g^−1^)	165.79 ± 37.53	164.85 ± 50.14	450.29 ± 164.43	623.84 ± 179.20
(DOPAC + HVA)/DA	4.11 ± 3.03	10.48 ± 28.02	2.01 ± 0.58	2.84 ± 0.81
MHPG/NA	0.11 ± 0.03	0.09 ± 0.03	0.03 ± 0.01	0.09 ± 0.02
5-HIAA/5-HT	0.22 ± 0.08	0.34 ± 0.09	0.38 ± 0.14	0.35 ± 0.09

*The turnover rates were calculated based on moles per milligram of the respective analyte*.

Ketoprofen attenuated the effects of LPS on serotonin (5-HT) concentrations in the hypothalamus [*F*_(3,47.14)_ = 2.86, *p* = 0.047; Figure 6], and 5-HT turnover rates tended to be affected [*F*_(3,47.79)_ = 0.99, *p* = 0.41, **Figure 8**]. SL pigs had significantly lower concentrations than KL pigs (*p* = 0.005) as reflected by higher turnover rates (*p* = 0.13). There were no differences between treatments and hemispheres in the frontal cortex, hippocampus, and brain stem.

**Figure 7:**
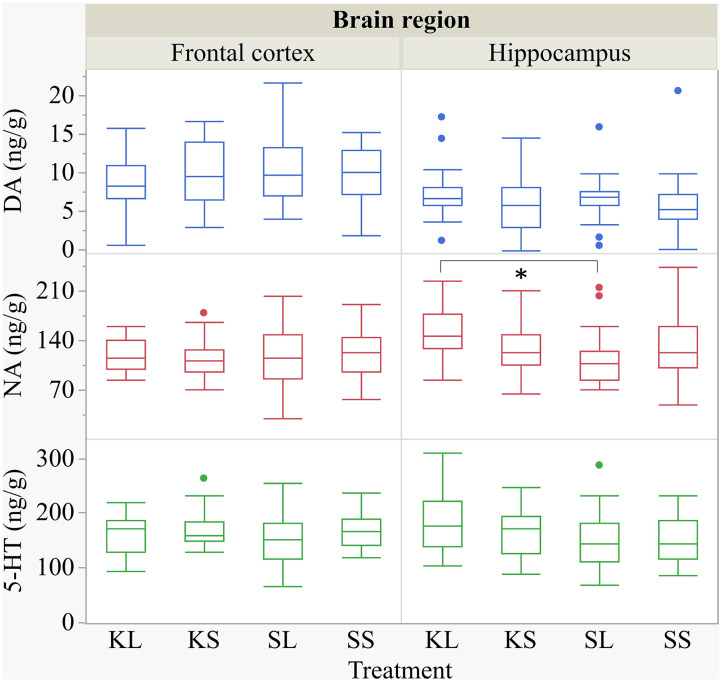
Concentration (ng g^−1^) of dopamine, noradrenaline, and serotonin in the frontal cortex and the hippocampus of pigs at 72 h after the injection with ketoprofen–lipopolysaccharide (LPS), ketoprofen–saline (KS), saline–LPS (SL), and saline–saline. To improve the readability, two outliers were removed from the figure (one KS in the frontal cortex and one SL in the hippocampus). Round dots indicate outliers. Significant differences of planned comparisons between treatments (*p* < 0.05) are marked with an asterisk.

**Figure 8:**
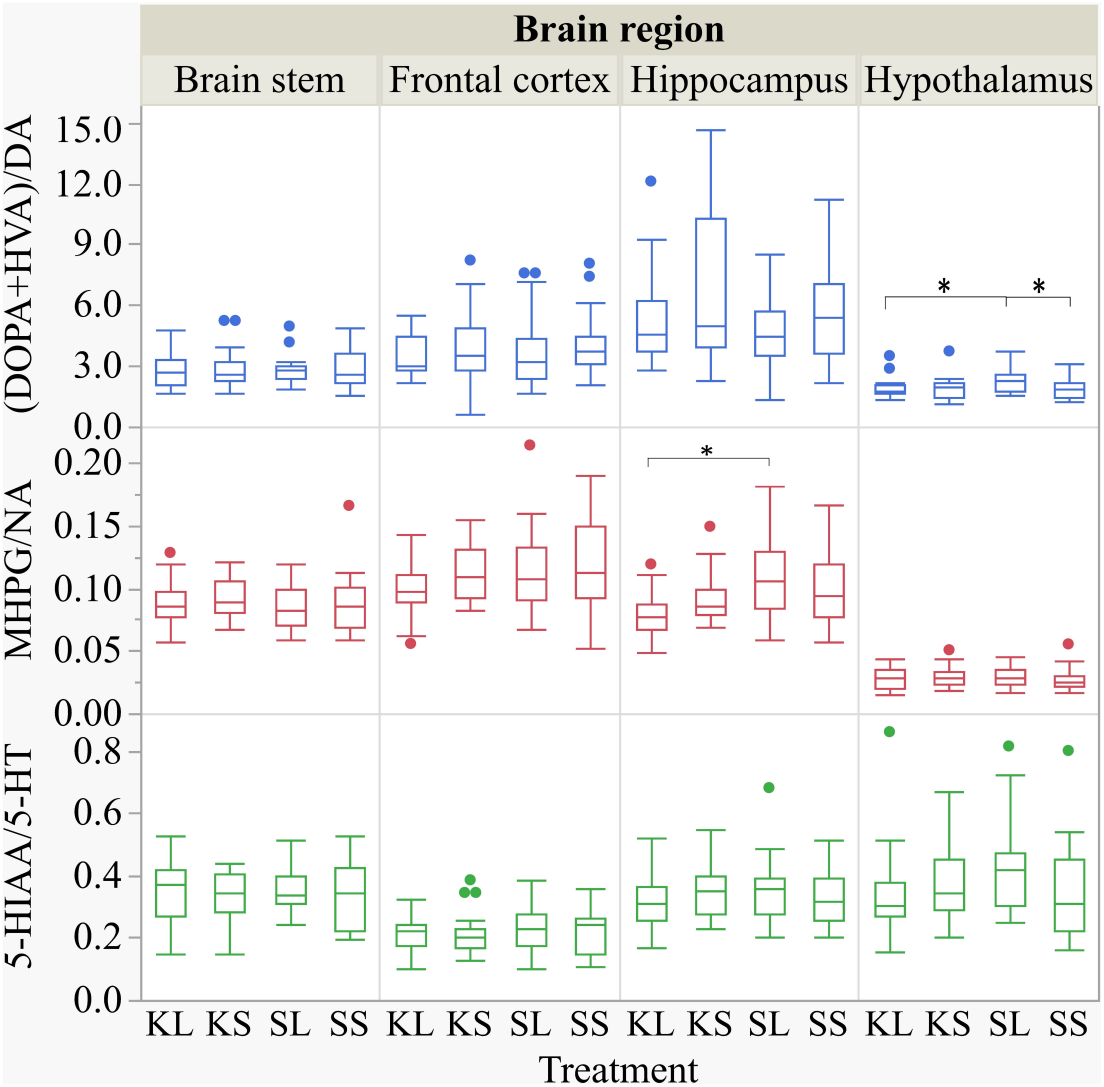
Dopamine turnover, noradrenaline turnover, and serotonin turnover in the brain stem, frontal cortex, hippocampus, and hypothalamus of pigs at 72 h after the injection with ketoprofen–lipopolysaccharide (LPS) (KL), ketoprofen–saline (KS), saline–LPS (SL), and saline–saline (SS). The turnover rates were calculated based on moles per milligram of the respective analyte. To improve the readability, seven outliers were removed from the figure (two KS, two SS, and two SL in the hippocampus and one KL in the frontal cortex). Round dots indicate outliers. Significant differences of planned comparisons between treatments (*p* < 0.05) are marked with an asterisk.

We found a similar result for NA concentrations in the hippocampus [*F*_(3,47.8)_ = 1.50, *p* = 0.23; Figure 7]. SL pigs had significantly lower concentrations compared to KL pigs (*p* = 0.04), which was reflected by significantly higher NA turnover rates [*F*_(3,47.73)_ = 2.96, *p* = 0.04; Figure 8] in SL pigs compared to KL pigs (*p* = 0.006). NA concentrations in the hypothalamus [*F*_(3,46.97)_ = 0.81, *p* = 0.49] tended to be lower in SL pigs compared to SS pigs (*p* = 0.13). There were no differences between treatments and hemispheres in the frontal cortex and brain stem. The covariate TIME had a significant effect on DA, 5-HT, and NA concentrations in the brain stem as reflected by decreasing concentrations with increasing time. All results of the analysis of variance are displayed in the Supplementary Tables E,F.

## Discussion

### Summary

LPS activated the HPA axis and elicited behavioral signs of sickness within 6 h after the injection as indicated by an increase in cortisol and decreased activity in LPS-injected pigs. Ketoprofen decreased the effect of LPS on cortisol release and alleviated behavioral signs of sickness. LPS had an effect on the duration of ear manipulation but not on any other behaviors. Controlled immune activation had no effect on the proinflammatory cytokines IFN-γ, TNF-α, and IL-18 measured at 72 h after the challenge in the frontal cortex, hippocampus, hypothalamus, and brain stem. The LPS-injected pigs had lower plasma concentrations of TRP at 72 h after the challenge compared to baseline. The correlations between TRP and KYN concentrations in plasma and brain tissue were weak and positive. The TRP and KYN concentrations were lower in the brain stem and frontal cortex of LPS-injected pigs compared to saline-injected pigs. The DA concentrations were lower in the hypothalamus of SL pigs compared to those in SS pigs. Ketoprofen attenuated the effects of LPS, resulting in lower concentrations of 5-HT in the hypothalamus and NA in the hippocampus of SL pigs compared to those in KL pigs.

### Effect of LPS and Ketoprofen on Behavioral Signs of Sickness and Social Behavior

LPS depressed activity in challenged pigs. As predicted, ketoprofen attenuated this effect. This observation corroborates the findings of Johnson and von Borell (29) who showed that treatment with indomethacin, another NSAID, completely inhibited anorexia as well as reduction in activity in LPS-injected pigs (5 μg kg^−1^, i.p.). Other studies showed that a pre-treatment of pigs with the NSAID meloxicam or flunixin significantly decreased the clinical symptoms induced by LPS (2–6 μg kg^−1^, i.v.), including lethargy, skin flushing, vomiting, coughing, and labored breathing (13, 30).

Most studies of LPS effects focus on a relatively short time period (24 h) and rarely describe social behavior in detail. In rodents, it was shown that, when sickness behavior resolves, mice display depressive-like behaviors measured by increased immobility in the forced swim test and tail suspension test as well as decreased sucrose preference up to 48 h after the challenge (10, 31–33). In the present study, LPS seemed not to have a strong impact on social interactions, and pre-treatment with ketoprofen neither had an effect in LPS-injected pigs nor in saline-injected controls. The LPS-injected pigs manipulated the ears of their pen mates significantly longer than the saline-injected pigs 2 days after the injection. Tail manipulation happened for a very short duration of time, and the variation was very high; thus, any possible differences would not have been evident in this rather small sample size. There are indications that the LPS-treated pigs experienced a long-term shift in social motivation, resulting in more tail- and ear-directed behavior compared to controls 2 days after the challenge (5). The relationship between sickness and altered social behavior was also found in the field where pigs diagnosed with respiratory diseases tended to perform more ear and tail biting than controls in the days before they were diagnosed as sick (34). There is a correlation between nosing the tail and tail biting and between nosing the ear and ear biting (35). Thus, our findings of more ear manipulation can be interpreted as a higher probability of damaging behavior. Nevertheless, severe tail, ear, and flank biting that result in wounds was not observed in the present study. This could be interpreted as an acute and short-lived immune activation that is handled well by pigs kept in the conditions that our pigs were kept in. These were rather optimal (small group sizes, low stocking density, organic enrichment) regarding other known risk factors for damaging behaviors. It has been reported that environmental enrichment modulates different aspects of the immune system and the acute phase response (36, 37). Moreover, the pigs were familiar with each other as they were kept in stable groups from birth on. Regrouping, which is common in commercial pig production, causes stress and activates the immune system as shown by elevated salivary cortisol (38) and induced leukocyte mobilization (39).

### Effect of LPS and Ketoprofen on HPA Axis Activity

LPS activates the HPA axis *via* prostaglandin (PGE2) release by endothelial cells as well as *via* a release of proinflammatory cytokines by macrophages. Salivary and plasma cortisols are highly correlated, and the values are around 10 times lower in saliva than in blood (40–42). In the present study, we collected saliva by letting pigs chew on a cotton pad. This is a non-invasive procedure compared to snaring the pigs for blood sampling which, in turn, has an effect on cortisol release by the adrenal glands. The significant elevation of salivary cortisol at 4 h after the challenge corroborates other studies measuring cortisol in plasma after intravenous (six) and intraperitoneal (20) LPS injection in pigs. The LPS-injected pigs that received a pre-treatment with ketoprofen showed a lower increase in salivary cortisol than SL pigs, but the concentrations were still higher than in SS pigs. Thus, a dose of 1.2 μg kg^−1^ LPS is sufficient to activate the HPA axis in 11- to 12-week-old pigs.

### Effect of LPS and Ketoprofen on Brain Cytokines

The rationale for including IFN-γ and TNF-α in the present study was mainly based on previous findings (6) and the relevance of these cytokines for kynurenine metabolism through induction of IDO (see “Section Effect of LPS and ketoprofen on plasma and brain concentrations of tryptophan and kynurenine”). IL-18 is, in turn, involved in IFN-γ production *via* NK cell activation which might serve as a bridge between innate and adaptive immunity (43).

In the present study, we did not monitor the short-term effects of LPS on cytokines in the blood, as this has been extensively studied in pigs in the past (6, 9, 13, 14). Our focus was beyond the first 24 h after the challenge, as we wanted to see if there were longer-lasting physiological changes. The duration of effect on physiology is important for the relevance of the LPS model in the study of immune effects on mental health and damaging behavior in pigs. If the physiological and behavioral changes are short-lived, they may not teach us much about the mechanisms underlying mental illness. In mice, it was shown that TNF-α concentrations in the brain remained elevated for 10 months after systemic LPS administration (44).

In the present study, LPS had no observed effect on the proinflammatory cytokines IFN-γ, TNF-α, and IL-18 in any of the sampled brain areas 3 days after the challenge. This finding contradicts with a previous study, where higher concentrations of IFN-y were found in the frontal cortex of LPS-injected pigs compared to saline-injected controls collected at the same time point (6). The proinflammatory cytokines in the brain are produced by microglia, the resident immune cells of the CNS [reviewed by Smith et al. (45)] or transported from blood. A single injection with a low dose of LPS might not be enough to activate microglia or to elevate brain cytokines over a period of 3 days.

LPS induces cytokine production through binding to TLR-4 and the subsequent activation of transcription factor NFkappaB (14). All NSAIDs inhibit the production of cyclooxygenases; in addition, some NSAIDs are able to alter the expression of NFkappaB (13) and thereby reduce cytokine expression. Plasma cytokine concentrations are usually not affected by the use of NSAIDs (13, 14), and there are few studies on brain cytokines in pigs so far. It was shown in mice that the use of NSAIDs (indomethacin and ibuprofen) reversed the effect of LPS on behavior without changing the peripheral or central cytokine concentrations (46). The absence of an effect of LPS on brain cytokines in the present study made it impossible to test an effect of ketoprofen on this.

### Effect of LPS and Ketoprofen on Plasma and Brain Concentrations of Tryptophan and Kynurenine

LPS influenced the tryptophan concentrations in plasma as well as the tryptophan and kynurenine concentrations in several brain areas. At 72 h, the LPS-injected pigs had lower plasma tryptophan concentrations compared to baseline, whereas the kynurenine concentrations were not affected. This corresponds to Melchior et al. (47), who demonstrated a depletion of tryptophan in plasma of pigs after experimentally induced lung inflammation over several days. In the short run, LPS caused a decrease in tryptophan and an increase in kynurenine serum concentrations in pigs measured at 3 h after the start of a 60-min LPS infusion (48). Wirthgen et al. (9) showed that tryptophan plasma concentrations were reduced for 24 h, whereas kynurenine concentrations were elevated for only 6 h after the LPS injection compared to the saline-injected pigs. It needs to be taken into account that SS pigs had a considerable baseline variation of plasma kynurenine concentrations, which might have led to a statistically significant but not biologically meaningful result.

In the present study, the effect of LPS on plasma tryptophan concentrations was not seen in pigs pre-treated with ketoprofen. This can be explained by the appetite-suppressant effect of IL-1, IL-6, IL-8, and TNF-α (49). The LPS-injected pigs showed depressed feeding and, in turn, reduced dietary uptake of tryptophan, which was reversed by ketoprofen. We found weak positive correlations between tryptophan concentrations in plasma and brain tissue. This effect was not seen in a study with mice where tryptophan concentrations were reduced in plasma but elevated in brain tissue at 24 h after the LPS injection (10).

The essential amino acid tryptophan is a peripheral precursor for the synthesis of the central neurotransmitter serotonin. Inflammation induces a shunt from serotonin to kynurenine metabolism of tryptophan mediated by the proinflammatory cytokines TNFα and IFNγ through an upregulation of the enzyme IDO. In the present study, we found lower tryptophan and kynurenine concentrations in the frontal cortex and brain stem as well as lower kynurenine concentrations in the hypothalamus of LPS-injected pigs compared to the saline-injected controls.

Ketoprofen had no influence on the effect of LPS on tryptophan and kynurenine concentrations in the sampled brain regions. There are few studies on tryptophan and kynurenine concentrations in the pig brain so far. In mice, the tryptophan concentrations in the hippocampus were found to be lower, and the kynurenine concentrations were higher 43 days after a chronic administration of LPS compared to the saline-treated controls (50). Kynurenine has been suggested by O'Connor et al. (10) to be essential for the development of depressive symptoms in rodents. As the changes in kynurenine concentrations last so shortly, subsequent changes in behavior might be caused by other mechanisms.

### Effect of LPS and Ketoprofen on Brain Monoamines

LPS has an effect on central neurotransmitters, which is supposed to be mediated by different proinflammatory cytokines such as IL-1, IL-6, and TNF-α [reviewed by Dunn (51)]. The LPS-injected pigs had lower dopamine concentrations and higher turnover rates in their hypothalamus compared to the saline-injected controls at 72 h after the challenge. Similar alterations were found in genetically stress-susceptible pigs (52). Dopamine in the hypothalamus is produced by cell groups that are concerned with the control of prolactin release and the regulation of preganglionic sympathetic neurons in the spinal cord (53). Apart from its relevance for the reward system, dopamine plays a key role in response to stress (54–56), and the hypothalamus is, as a part of the HPA axis, one of the two major systems that respond to stress.

The intervention with ketoprofen alleviated the effects of LPS on serotonin in the hypothalamus and noradrenaline and its turnover rate in the hippocampus. The most important source of central noradrenaline, which is synthetized among the same pathways as dopamine, is the clusters of cell bodies in the locus coeruleus located in the pons that send branching axons, among others, to the hippocampus (57). The hippocampus plays a role in the control of the HPA response to stress and is itself an important target for glucocorticoids (58). The neurotransmitter serotonin derives from cell bodies in the raphe nuclei in the pons (part of the brain stem) that projects, among others, to the hypothalamus and the hippocampus (57) and has modulatory effects in almost all central nervous system integrative functions such as stress and fearfulness (59), aggression (60, 61), and mood. There is a close relationship between mood and state of arousal. In mice, lower concentrations of noradrenaline and serotonin in the prefrontal cortex (8) and lower levels of serotonin in the hippocampus of LPS-injected mice compared to controls were found at 24 h after the challenge (7). In both studies, depressive-like behaviors in forced swim and tail suspension tests were observed in LPS-injected mice. It needs to be taken into account that the focus of the present study is on (negative) social behavior as there is no validated model for depressive-like behaviors in pigs. In rats, a pre-treatment with the NSAID diclofenac completely prevented IFN-α-increased serotonin turnover in the prefrontal cortex and increased dopamine turnover in the hippocampus (62). A pre-treatment with the NSAID indomethacin attenuated but did not abolished the increased extracellular levels of serotonin and noradrenaline in the hippocampus of rats within 6 h after the LPS challenge (63).

## Conclusion

A controlled immune activation altered the behavior of the pigs in the 1st hours after the challenge but seemed not to have a strong impact on their social interactions in the subsequent 2 days. Central cytokine concentrations were not elevated 3 days after the challenge, but central monoamines were downregulated, and plasma tryptophan was depleted in LPS-injected pigs. Ketoprofen attenuated the effects of immune activation on behavioral signs of sickness, HPA axis activity, and central monoamines. LPS at a dose of 1.2 μg kg^−1^ seems not to be applicable as a model for long-term changes in the social behavior of pigs.

## Data Availability Statement

The raw data supporting the conclusions of this article will be made available by the authors, without undue reservation.

## Ethics Statement

The animal study was reviewed and approved by the NMBU IACUC and the food safety authorities (FOTS ID 15232).

## Author Contributions

Research idea and experimental design: JN, AMJ, and AV. Conduction of the experiment: CV, AMJ, JN, and BR. Behavioral observation: CV and JV supervised by AV and JN. Labwork: PP, DD, and CV. Data analysis: CV supervised by JN. Interpretation of data: all authors. Drafting the publication: CV. Revision of publication: all authors.

## Conflict of Interest

The authors declare that the research was conducted in the absence of any commercial or financial relationships that could be construed as a potential conflict of interest.
