# Supplementary material for: The Effect of LPS and Ketoprofen on Cytokines, Brain Monoamines, and Social Behavior in Group-Housed Pigs
Source: Front Vet Sci. 2021 Jan 7;7:617634. doi: 10.3389/fvets.2020.617634 (PMC7873924; doi:10.3389/fvets.2020.617634)
Supplement: Supplementary file 1 [file Table_1.DOCX]

Table A: Frequency and duration [s] of all performed and received behaviours observed during 72 h after injection with ketoprofen-LPS (KL), ketoprofen-saline (KS), saline-LPS (SL) and saline-saline (SS).

|  | | | **KL** | | | **KS** | | | **SL** | | | **SS** | | |
| --- | --- | --- | --- | --- | --- | --- | --- | --- | --- | --- | --- | --- | --- | --- |
|  |  |  | **DAY0** | **DAY2** | **DAY3** | **DAY0** | **DAY2** | **DAY3** | **DAY0** | **DAY2** | **DAY3** | **DAY0** | **DAY2** | **DAY3** |
| **Tail manipulation** | performed | Duration, median (min\|max) | 4.94 (0\|298.68) | 16.12 (0\|117) | 10.46 (0\|37.68) | 38.88 (0\|79.76) | 14.26 (0\|55.2) | 10.58 (0\|48.08) | 20.92 (0\|125.8) | 13.78 (0\|62.64) | 3.9 (0\|31.64) | 11.1 (0\|240.4) | 6.54 (0\|276.48) | 5.8 (0\|35.88) |
|  |  | Frequency, median (min\|max) | 1  (0\|4) | 2.5  (0\|6) | 1.5  (0\|4) | 3.5 (0\|9) | 1.5 (0\|6) | 3 (0\|4) | 1.5 (0\|14) | 1.5 (0\|11) | 1.5 (0\|5) | 3 (0\|10) | 2 (0\|22) | 1 (0\|5) |
|  | received | Duration, median (min\|max) | 27.72 (7.56\|146.32) | 19.94 (0\|84.48) | 16.76 (0\|63.2) | 11.72 (0\|76.72) | 17.94 (0\|39.84) | 4.44 (0\|37.76) | 10.26 (0\|319.92) | 15.48 (0\|88.52) | 29.42 (0\|70.88) | 33.96 (4.84\|133.68) | 21.22 (0\|74.84) | 14.82 (0\|59.68) |
|  |  | Frequency, median (min\|max) | 3 (1\|10) | 2.5 (0\|10) | 2.5 (0\|5) | 1 (0\|10) | 2.5 (0\|7) | 1 (0\|5) | 2.5 (0\|8) | 2.5 (0\|8) | 3 (0\|7) | 3 (1\|10) | 2.5 (0\|9) | 2.5 (0\|5) |
| **Ear manipulation** | performed | Duration, median (min\|max) | 55.4 (0\|306.8) | 9.76 (0\|612.36) | 50.92 (4.04\|207) | 56.42 (0\|274.24) | 54.2(13.56\|344.48) | 84.22 (6.32\|405.48) | 49.74 (0\|592.24) | 95.56 (0\|447.92) | 118.82 (0\|160.24) | 27.88 (0\|160.24) | 62.16 (7.4\|362.28) | 37.38 (0\|97.72) |
|  |  | Frequency, median (min\|max) | 5.5 (0\|35) | 2 (0\|38) | 6 (1\|13) | 5.5 (0\|15) | 5.5 (2\|18) | 6.5 (2\|18) | 7.5 (0\|31) | 8 (0\|32) | 5 (0\|26) | 3.5 (0\|17) | 8.5 (2\|18) | 4 (0\|7) |
|  | received | Duration, median (min\|max) | 66.1 (7.32\|196.68) | 50.48 (8\|208.76) | 71.6 (0\|180.08) | 71.02 (19.88\|190.12) | 55.88 (19.6\|346.64) | 101.6 (5.68\|281.24) | 37.78 (0\|199.12) | 60.68 (27.72\|232.6) | 39.06 (4.16\|147.2) | 46.78 (20.16\|137.44) | 52.68 (11.04\|286.88) | 83.6 (18.24\|165.48) |
|  |  | Frequency, median (min\|max) | 6 (1\|19) | 5.5 (2\|12) | 5 (0\|13) | 7.5 (2\|15) | 7.5 (3\|14) | 8.5 (2\|15) | 4 (0\|14) | 8 (3\|11) | 5 (1\|9) | 6.5 (2\|17) | 5.5 (2\|18) | 7.5 (2\|22) |
| **Manipulation of other body parts** | performed | Duration, median (min\|max) | 73.18 (7.76\|149.36) | 62.48 (10.04\|174.52) | 79.04 (11.96\|271.04) | 97.96 (10.8\|311.88) | 107.52 (37.76\|229.64) | 151.36 (12.24\|230.44) | 61.84 (4.96\|223.6) | 51.12 (6.4\|630.76) | 63.96 (14.84\|510.36) | 66.86 (2.88\|378.83) | 68.52 (2.8\|370.44) | 69.34 (12.96\|302.24) |
|  |  | Frequency, median (min\|max) | 8.5 (1\|19) | 9 (2\|16) | 10.5 (3\|24) | 10 (1\|27) | 9.5 (4\|18) | 9.5 (3\|22) | 8.5 (0\|28) | 8.5 (1\|65) | 7.5 (1\|35) | 5.5 (0\|29) | 8 (1\|18) | 7 (1\|16) |
|  | received | Duration, median (min\|max) | 112.4 (22.52\|331) | 93.54 (0\|266.16) | 80.86 (6.44\|164.6) | 77.7 (18.72\|157.36) | 79.22 (28.64\|295.96) | 102.84 (38.44\|216.52) | 90.78 (0\|232.8) | 69.5 (10.72\|229.32 | 59.9 (0\|282.72) | 102 (23.44\|275.6) | 82.22 (2.2\|276.52) | 95.12 (13\|487.72) |
|  |  | Frequency, median (min\|max) | 8.5 (3\|23) | 9 (0\|27) | 7.5 (2\|14) | 9 (3\|20) | 8 (4\|17) | 8.5 (4\|15) | 9 (0\|19) | 7.5 (2\|15) | 8 (0\|24) | 9.5 (3\|17) | 8.5 (1\|32) | 8 (3\|28) |
| **Fighting** | performed | Duration, median (min\|max) | 12 (0\|57) | 35.2 (0\|68.16) | 19.24 (0\|44.24) | 30.38 (7\|89.36) | 27.93 (0\|91.56) | 27.3 (0\|172.52) | 21.08 (2\|74) | 34.08 (0\|65.44) | 12.14 (0\|139.52) | 13 (0\|82.92) | 50.28 (0\|299.36) | 17.64 (0\|76.2) |
|  |  | Frequency, median (min\|max) | 3 (0\|13) | 7.5 (0\|16) | 5.5 (0\|14) | 6 (1\|12) | 6 (0\|21) | 5.5 (0\|27) | 5 (1\|16) | 5 (0\|12) | 3 (0\|33) | 3 (0\|12) | 6.5 (0\|31) | 4 (0\|13) |
|  | received | Duration, median (min\|max) | 19.2 (1.8\|83.96) | 16.64 (5.58\|121.96) | 27.36 (2.96\|84.24) | 20.16 (0\|146.68) | 46.98 (0\|129) | 38.16 (8.84\|99.44) | 36.2 (6.12\|114.96) | 33.9 (8.36\|59.16) | 20.32 (5.84\|130.16) | 15.92 (0\|65.96) | 36.82 (0\|89.92) | 28.7 (0\|72.16) |
|  |  | Frequency, median (min\|max) | 4.5 (1\|15) | 4.5 (1\|21) | 9.5 (2\|15) | 6 (0\|14) | 11 (0\|23) | 8 (1\|16) | 9 (2\|19) | 5.5 (2\|19) | 5 (1\|25) | 4.5 (0\|17) | 5 (0\|18) | 6 (0\|16) |
| **Displacement** | performed | Duration, median (min\|max) | 0 (0\|9.08) | 0 (0\|15) | 1 (0\|17.36) | 0 (0\|19.12) | 0 (0\|20.24) | 0 (0\|20.88) | 0 (0\|13.2) | 0 (0\|13.64) | 0 (0\|9.2) | 0 (0\|24.16) | 0 (0\|18.12) | 0 (0\|7.88) |
|  |  | Frequency, median (min\|max) | 0 (0\|2) | 0 (0\|5) | 0.5 (0\|4) | 0 (0\|5) | 0 (0\|6) | 0 (0\|4) | 0 (0\|3) | 0 (0\|4) | 0 (0\|3) | 0 (0\|6) | 0 (0\|5) | 0 (0\|2) |
|  | received | Duration, median (min\|max) | 0.9 (0\|9.36) | 0 (0\|27) | 0 (0\|7.84) | 0 (0\|23.12) | 1.6 (0\|17.4) | 0 (0\|19.56) | 0 (0\|10.8) | 0 (0\|13.48) | 0 (0\|6.441) | (0\|14.24) | 0 (0\|8.44) | 0 (0\|10.68) |
|  |  | Frequency, median (min\|max) | 0.5 (0\|2) | 0 (0\|4) | 0 (0\|2) | 0 (0\|6) | 0.5 (0\|4) | 0 (0\|4) | 0 (0\|3) | 0 (0\|4) | 0 (0\|1) | 0.5 (0\|3) | 0 (0\|3) | 0 (0\|2) |
| **Flank nosing** | performed | Duration, median (min\|max) | 4.5 (0\|52) | 3.9 (0\|25) | 3 (0\|45) | 5.5 (0\|56) | 8.84 (0\|49) | 0 (0\|112) | 10.38 (0\|41) | 2 (0\|41) | 0 (0\|120) | 9 (0\|30) | 1.5 (0\|167.96) | 10.5 (0\|96) |
|  |  | Frequency, median (min\|max) | 1 (0\|4) | 1 (0\|2) | 1 (0\|5) | 1 (0\|8) | 1 (0\|6) | 0 (0\|10) | 1 (0\|5) | 0.5 (0\|6) | 0 (0\|7) | 2 (0\|3) | 0.5 (0\|5) | 1 (0\|6) |
|  | received | Duration, median (min\|max) | 7.28 (0\|44.44) | 3.42 (0\|138.08) | 8.64 (0\|103.04) | 10.22 (0\|65.4) | 1.6 (0\|76.16) | 0.12 (0\|47.08) | 2.34 (0\|41.56) | 2.28 (0\|24.64) | 2.52 (0\|609.6) | 2.12 (0\|214.96) | 3.22 (0\|244) | 0 (0\|166.2) |
|  |  | Frequency, median (min\|max) | 1.5 (0\|7) | 1 (0\|3) | 1 (0\|6) | 1 (0\|5) | 0.62 (0\|4) | 0.12 (0\|5) | 1 (0\|6) | 0.62 (0\|3) | 1 (0\|6) | 1 (0\|4) | 0.5 (0\|7) | 0 (0\|7) |
| **Belly nosing** | performed | Duration, median (min\|max) | 4.48 (0\|268) | 0 (0\|136) | 2 (0\|591) | 3.56 (0\|306) | 0 (0\|74) | 1.08 (0\|268) | 0 (0\|17.84) | 0 (0\|116) | 0 (0\|201) | 0 (0\|321) | 0.5 (0\|248) | 0 (0\|239) |
|  |  | Frequency, median (min\|max) | 1 (0\|8) | 0 (0\|5) | 1 (0\|5) | 1 (0\|4) | 0 (0\|4) | 1(0\|4) | 0 (0\|2) | 0 (0\|13) | 0 (0\|7) | 0 (0\|5) | 0.5 (0\|3) | 0 (0\|11) |
|  | received | Duration, median (min\|max) | 3.26 (0\|171.04) | 0 (0\|314.12) | 1.04 (0\|82.44) | 2.78 (0\|200.16) | 1.48 (0\|105.52) | 0 (0\|684.68) | 2.2 (0\|152.64) | 2.72 (0\|350.88) | 0.54 (0\|53.28) | 0 (0\|644.64) | 0 (0\|151.92) | 0 (0\|273.4) |
|  |  | Frequency, median (min\|max) | 1 (0\|5) | 0 (0\|4) | 1 (0\|6) | 1 (0\|4) | 0.5 (0\|6) | 0 (0\|10) | 0.5 (0\|5) | 0.5 (0\|5) | 0.5 (0\|2) | 0 (0\|11) | 0 (0\|7) | 0 (0\|7) |
