# Supplementary material for: The Effect of LPS and Ketoprofen on Cytokines, Brain Monoamines, and Social Behavior in Group-Housed Pigs
Source: Front Vet Sci. 2021 Jan 7;7:617634. doi: 10.3389/fvets.2020.617634 (PMC7873924; doi:10.3389/fvets.2020.617634)
Supplement: Supplementary file 2 [file Table_2.DOCX]

Table B: Results of the analysis of variance (ANOVA) of duration and frequency of performed and received behaviour for the interaction of treatment and day.

| Behaviour |  | ANOVA | F(treatment*day) | p-value |
| --- | --- | --- | --- | --- |
| Tail manipulation | performed | Duration | F_6,87.58_ = 0.23 | 0.97 |
|  |  | Frequency | F_6,88_ = 0.83 | 0.55 |
|  | received | Duration | F_6,88_ = 0.70 | 0.65 |
|  |  | Frequency | F_6,88_ = 0.83 | 0.55 |
| Ear manipulation | performed | Duration | F_6,88_ = 1.49 | 0.19 |
|  |  | Frequency | F_6,88_ = 1.38 | 0.23 |
|  | received | Duration | F_6,88_ = 0.48 | 0.82 |
|  |  | Frequency | F_6,88_ = 1.74 | 0.12 |
| Flank nosing | performed | Duration | F_6,88_ = 0.20 | 0.98 |
|  |  | Frequency | F_6,88_ = 0.35 | 0.91 |
|  | received | Duration | F_6,88_ = 0.85 | 0.54 |
|  |  | Frequency | F_6,88_ = 0.39 | 0.88 |
| Belly nosing | performed | Duration | F_6,88_ = 0.33 | 0.92 |
|  |  | Frequency | F_6,88_ = 0.54 | 0.78 |
|  | received | Duration | F_6,88.57_ = 0.72 | 0.63 |
|  |  | Frequency | F_6,88.59_ = 0.26 | 0.95 |
| Manipulation of other body parts | performed | Duration | F_6,88_ = 0.36 | 0.90 |
|  |  | Frequency | F_6,88_ = 0.10 | 1.00 |
|  | received | Duration | F_6,88_ = 0.77 | 0.60 |
|  |  | Frequency | F_6,88_ = 0.12 | 0.99 |
| Fighting | performed | Duration | F_6,88_ = 0.86 | 0.53 |
|  |  | Frequency | F_6,88_ = 0.54 | 0.78 |
|  | received | Duration | F_6,88_ = 0.42 | 0.87 |
|  |  | Frequency | F_6,88_ = 0.38 | 0.89 |
| Displacement | performed | Duration | F_6,88_ = 1.00 | 0.43 |
|  |  | Frequency | F_6,88_ = 0.87 | 0.52 |
|  | received | Duration | F_6,88_ = 0.78 | 0.59 |
|  |  | Frequency | F_6,88_ = 0.95 | 0.46 |
