# Supplementary material for: The Effect of LPS and Ketoprofen on Cytokines, Brain Monoamines, and Social Behavior in Group-Housed Pigs
Source: Front Vet Sci. 2021 Jan 7;7:617634. doi: 10.3389/fvets.2020.617634 (PMC7873924; doi:10.3389/fvets.2020.617634)
Supplement: Supplementary file 3 [file Table_3.DOCX]

Table C: Results of the analysis of variance (ANOVA) of interferon gamma (IFN-γ), tumor necrosis factor alpha (TNF-α) and interleukin 18 (IL-18) for treatment and hemisphere according to brain region.

| Brain region | ANOVA | IFN-γ | | TNF-α | | IL-18 | |
| --- | --- | --- | --- | --- | --- | --- | --- |
|  |  | **F-ratio** | **p-value** | **F-ratio** | **p-value** | **F-ratio** | **p-value** |
| Frontal cortex | Treatment  Hemisphere | F_(3,48.67)_ = 0.34  F_(1,50.62)_ = 0.0004 | 0.79  0.98 | F_(3,46,.6)_ = 0.98  F_(1,50.64)_ = 2.60 | 0.41  0.11 | F_(3,48,32)_ = 0.45  F_(1,48.57)_ = 0.68 | 0.72  0.41 |
| Hippo-campus | Treatment  Hemisphere | F_(3,48.75)_ = 1.04  F_1,50.49_ = 0.91 | 0.38  0.34 | F_(3,48.11)_ = 1.32  F_(1,50.29)_ = 0.99 | 0.28  0.33 | F_(3,48.22)_ = 0.53  F_(1,48.73)_ = 0.12 | 0.66  0.73 |
| Hypo-thalamus | Treatment  Hemisphere | F_(3,46)_ = 0.24^a^  F_(1,48.64)_ = 0.65^a^ | 0.87  0.42 | F_(3,48.31)_ = 0.12^a^ F_(1,54.21)_ = 0.18^a^ | 0.95  0.67 | F_(3,47.76)_ = 0.93  F_(1,47.62)_ = 0.12 | 0.43  0.73 |
| Brain stem | Treatment  Hemisphere | F_(3,47)_ = 0.14^a^  F_(1,51)_ = 3.23^a^ | 0.94  0.08 | F_(3,48)_ = 0.91  F_(1,51)_ = 5.51 | 0.44  0.02* | F_(3,48)_ = 2.01  F_(1,51)_ = 0.02 | 0.13  0.88 |

Significant results (p < 0.05) are marked with *

^a^Covariate TIME included in the model
