# Supplementary material for: The Effect of LPS and Ketoprofen on Cytokines, Brain Monoamines, and Social Behavior in Group-Housed Pigs
Source: Front Vet Sci. 2021 Jan 7;7:617634. doi: 10.3389/fvets.2020.617634 (PMC7873924; doi:10.3389/fvets.2020.617634)
Supplement: Supplementary file 4 [file Table_4.DOCX]

| Brain region | ANOVA | TRP | | KYN | | KYN/TRP | |
| --- | --- | --- | --- | --- | --- | --- | --- |
|  |  | **F-ratio** | **p-value** | **F-ratio** | **p-value** | **F-ratio** | **p-value** |
| Frontal cortex | Treatment  Hemisphere | F_(3,45.41)_ = 2.04^a^  F_(1,46.16)_ = 0.12^a^ | 0.12  0.73 | F_(3,47.4)_ = 4.0^a^ F_(1,48.83)_ = 2.8^a^ | 0.01*  0.10 | F_(3,47.02)_ = 1.95^a^  F_(1,48.63)_ = 2.85^a^ | 0.13  0.10 |
| Hippo-campus | Treatment  Hemisphere | F_(3,46.52)_ = 1.37^a^  F_(1,47.92)_ = 3.86^a^ | 0.26  0.06 | F_(3,46.99)_ = 1.31^a^  F_(1,48.5)_ = 0.40^a^ | 0.28  0.53 | F_(3,47.17)_ = 0.68^a^  F_(1,48.97)_ = 1.25^a^ | 0.57  0.27 |
| Hypo-thalamus | Treatment  Hemisphere | F_(3,44.39)_ = 0.73^a^  F_(1,45.82)_ = 3.33^a^ | 0.54  0.07 | F_(3,47.11)_ = 2.43 F_(1,45.51)_ = 0.12 | 0.08  0.73 | F_(3,46.76)_ = 2.21  F_(1,44.83)_ = 2.06 | 0.10  0.16 |
| Brain stem | Treatment  Hemisphere | F_(3,47.94)_ = 1.90  F_(1,50.2)_ = 0.33 | 0.14  0.57 | F_(3,48.24)_ = 2.33  F_(1,50.61)_ = 1.58 | 0.09  0.21 | F_(3,48.25)_ = 1.81  F_(1,50.62)_ = 0.94 | 0.16  0.34 |

Table D: Results of the analysis of variance (ANOVA) of tryptophan (TRP), kynurenine (KYN) and kynurenine-tryptophan ratio (KYN/TRY) for treatment and hemisphere according to brain region.

Significant results (p < 0.05) are marked with *

^a^Covariate TIME included in the model
