# Supplementary material for: The Effect of LPS and Ketoprofen on Cytokines, Brain Monoamines, and Social Behavior in Group-Housed Pigs
Source: Front Vet Sci. 2021 Jan 7;7:617634. doi: 10.3389/fvets.2020.617634 (PMC7873924; doi:10.3389/fvets.2020.617634)
Supplement: Supplementary file 5 [file Table_5.DOCX]

Table E: Results of the analysis of variance (ANOVA) of dopamine (DA), noradrenaline (NA), serotonin (5-HT) for treatment and hemisphere according to brain region.

| Brain region | ANOVA | DA | | NA | | 5-HT | |
| --- | --- | --- | --- | --- | --- | --- | --- |
|  |  | **F-ratio** | **p-value** | **F-ratio** | **p-value** | **F-ratio** | **p-value** |
| Frontal cortex  n = 103 | Treatment  Hemisphere | F_(3,48.53)_ = 0.83  F_(1,51.41)_ = 0.89 | 0.48  0.35 | F_(3,47.25)_ = 0.11  F_(1,47.15)_ = 0.11 | 0.95  0.75 | F_(3,47.32)_ = 0.60  F_(1,47.14)_ = 1.40 | 0.62  0.24 |
| Hippo-campus  n = 102 | Treatment  Hemisphere | F_(3,47.47)_ = 0.97  F_(1,49.69)_ = 0.08 | 0.42  0.78 | F_(3,47.80)_ = 1.50  F_(1,48.37)_ = 0.29 | 0.23  0.59 | F_(3,47.77)_ = 1.07  F_(1,48.41)_ = 0.21 | 0.37  0.65 |
| Hypo-thalamus  n = 93 | Treatment  Hemisphere | F_(3,47.06)_ = 2.05  F_(1,48.19)_ = 0.003 | 0.12  0.95 | F_(3,46.97)_ = 0.81  F_(1,46.55)_ = 2.20 | 0.49  0.15 | F_(3,47.14)_ = 2.86  F_(1,47.52)_ = 3.98 | 0.047*  0.05 |
| Brain stem  n = 104 | Treatment  Hemisphere | F_(3,47.34)_ = 0.30^a^  F_(1,50.87)_ = 2.27^a^ | 0.83  0.14 | F_(3,47.24)_ = 0.95^a^  F_(1,50.46)_ = 0.99^a^ | 0.43  0.33 | F_(3,47.13)_ = 0.11^a^  F_(1,50.4)_ = 3.61^a^ | 0.96  0.06 |

Significant results (p < 0.05) are marked with *

^a^Covariate TIME included in the model
