# Supplementary material for: The Effect of LPS and Ketoprofen on Cytokines, Brain Monoamines, and Social Behavior in Group-Housed Pigs
Source: Front Vet Sci. 2021 Jan 7;7:617634. doi: 10.3389/fvets.2020.617634 (PMC7873924; doi:10.3389/fvets.2020.617634)
Supplement: Supplementary file 6 [file Table_6.DOCX]

| Brain region | ANOVA | (DOPAC+HVA/DA) | | MHPG/NA | | 5-HIAA  /5-HT | |
| --- | --- | --- | --- | --- | --- | --- | --- |
|  |  | **F-ratio** | **p-value** | **F-ratio** | **p-value** | **F-ratio** | **p-value** |
| Frontal cortex  n = 103 | Treatment  Hemisphere | F_(3,47.9)_ = 0.32  F_(1,51.02)_ = 0.56 | 0.81  0.46 | F_(3,48.76)_ = 0.98  F_(1,49.23)_ = 0.0005 | 0.41  0.98 | F_(3,48.63)_ = 0.21  F_(1,50.08)_ = 0.53 | 0.89  0.47 |
| Hippo-campus  n = 102 | Treatment  Hemisphere | F_(3,48.94)_ = 0.66  F_(1,50.98)_ = 0.05 | 0.58  0.83 | F_(3,47.73)_ = 2.95  F_(1,48.69)_ = 5.46 | 0.04*  0.02* | F_(3,46.58)_ = 0.78  F_(1,47.81)_ = 1.65 | 0.51  0.21 |
| Hypo-thalamus  n = 93 | Treatment  Hemisphere | F_(3,46.16)_ = 2.28  F_(1,46.26)_ = 0.008 | 0.09  0.92 | F_(3,47.02)_ = 0.30  F_(1,47.01)_ = 0.44 | 0.83  0.51 | F_(3,47.79)_ = 0.99  F_(1,47.22)_ = 0.82 | 0.41  0.37 |
| Brain stem  n = 104 | Treatment  Hemisphere | F_(3,47.84)_ = 0.02  F_(1,50.13)_ = 1.58 | 1.00  0.21 | F_(3,48.16)_ = 0.28  F_(1,50.38)_ = 0.004 | 0.84  0.95 | F_(3,48.1)_ = 0.33  F_(1,50.38)_ = 1.02 | 0.80  0.32 |

Table F: Results of the analysis of variance (ANOVA) of dopamine turnover (DOPAC+HVA/DA), noradrenaline turnover (MHPG/NA), serotonin turnover (5-HIAA/5-HT) for treatment and hemisphere according to brain region. Turnover rates were calculated based on mol mg^-1^ of the respective analyte.

Significant results (p < 0.05) are marked with *
